# Graph-Aware and Sequence-Aware Multimodal Deep Learning Framework for Cancer Detection and Risk Analysis from Medical Imaging

**DOI:** 10.3390/jimaging12090431

**Published:** 2026-09-10

**Authors:** Chetanpal Singh, Santoso Wibowo, Srimannarayana Grandhi, Satria Mandala

**Affiliations:** 1 School of Engineering and Technology, Central Queensland University, Melbourne 3000, Australia; s.wibowo1@cqu.edu.au (S.W.); s.grandhi@cqu.edu.au (S.G.); 2 Human Centric Engineering, Telkom University, Bandung 40257, Indonesia; satriamandala@telkomuniversity.ac.id

**Keywords:** deep learning, medical image analysis, graph attention network, multimodal learning, sequence-aware learning, breast cancer classification, lung nodule classification, cross-attention fusion, explainable artificial intelligence, computer-aided diagnosis

## Abstract

Early and accurate cancer detection from medical imaging remains challenging because clinically relevant evidence is distributed across local image appearance, structural relationships between suspicious regions, and ordered imaging context. This study proposes a graph-aware and sequence-aware deep learning framework that combines a convolutional neural network (CNN) backbone for spatial feature extraction, a Graph Attention Network (GAT) for lesion-structure modelling, and a Bidirectional Long Short-Term Memory (BiLSTM) module for ordered-view or slice-sequence representation learning. Cross-attention-based multimodal fusion is evaluated exclusively for the RSNA mammography task, where the metadata branch is restricted to patient age and implant status, both available before diagnosis. In contrast, the primary LIDC-IDRI experiment is conducted as an image-only analysis because radiologist malignancy scores and semantic nodule attributes are annotation-derived variables and are not treated as independent clinical predictors. The framework is evaluated on the RSNA Breast Cancer Detection dataset and the LIDC-IDRI lung CT dataset using accuracy, precision, recall, F1-score, specificity, and area under the receiver operating characteristic curve (AUC). Additional ablation experiments assess the contribution of graph learning, sequence-aware modelling, and leakage-safe RSNA metadata fusion, while SHAP analysis quantifies the influence of the included RSNA metadata variables on multimodal predictions. For the RSNA multimodal experiment, the best held-out run achieved 95.2% accuracy and an AUC of 0.978, while three repeated runs yielded 94.0 ± 0.3% accuracy and an AUC of 0.970 ± 0.007 under the internal patient-wise benchmark protocol. These results should be interpreted as public-dataset benchmark outcomes rather than evidence of real-world clinical performance; external multi-centre and prospective validation is required before clinical deployment.

## 1. Introduction

Cancer remains one of the leading causes of mortality worldwide, with early detection playing a critical role in improving patient survival and treatment outcomes [1]. Medical imaging modalities such as mammography, computed tomography (CT), and magnetic resonance imaging (MRI) are widely used for screening and diagnosis. However, accurately identifying early-stage malignancies is challenging due to subtle visual patterns, heterogeneous tumour structures and variability across patients and imaging conditions. In addition, tumour development is inherently dynamic, evolving over time, which further complicates diagnosis when images are analysed independently rather than as part of an ordered imaging sequence.

Recent advances in deep learning have significantly improved medical image analysis, particularly through convolutional neural networks (CNNs), which are capable of automatically learning hierarchical feature representations from raw imaging data. CNN-based approaches have demonstrated strong performance in tumour detection, segmentation, and classification tasks across multiple imaging modalities [1,2]. Despite these advancements, conventional CNN models operate on grid-based image representations and are limited in their ability to capture complex spatial relationships between tumour regions and surrounding tissues. Such relationships are often clinically important, as tumour growth and malignancy are influenced by interactions with adjacent anatomical structures.

To overcome these limitations, attention mechanisms have been introduced to enhance feature representation by allowing models to focus on the most relevant regions within an image. Attention-based deep learning models have shown improved performance by dynamically weighting features according to their importance [3,4]. Prior work has demonstrated that integrating attention layers with sequence models such as Long Short-Term Memory (LSTM) can significantly enhance feature weighting and classification accuracy. However, these approaches still treat spatial features independently and do not explicitly model structural dependencies within the image.

Graph Neural Networks (GNNs) provide a powerful framework for modelling non-Euclidean data and have recently been applied to medical imaging to represent relationships between regions of interest. By modelling images as graphs, where nodes correspond to image patches or anatomical regions and edges represent spatial or semantic relationships, GNNs enable the capture of complex structural patterns that are not accessible through traditional CNN architectures [5,6]. Graph Attention Networks (GATs) allow adaptive weighting of neighbouring nodes, improving both performance and interpretability.

Although true temporal learning is valuable in clinical oncology, the datasets used in this study do not contain longitudinal follow-up scans with verified disease evolution labels [7]. Therefore, this manuscript uses the BiLSTM module for sequence-aware representation learning rather than for confirmed longitudinal progression modelling. For RSNA mammography, the ordered inputs are complementary mammographic views such as CC and MLO views. For LIDC-IDRI, the ordered inputs are neighbouring CT slices around a nodule, representing volumetric continuity. This distinction is important because it prevents overclaiming while still allowing the model to learn meaningful dependencies across ordered imaging representations [8,9,10].

In addition to imaging data, clinical decision-making often depends on contextual information such as patient demographics, medical history, and diagnostic reports. However, many existing deep learning models are unimodal and fail to integrate such auxiliary data effectively. Multimodal learning approaches aim to combine heterogeneous data sources to improve predictive performance. Cross-attention mechanisms, inspired by transformer architectures, have shown strong capability in aligning and fusing information from multiple modalities by learning interactions between them [9].

Motivated by these challenges, this study proposes a graph-aware and sequence-aware deep learning framework for cancer detection and risk analysis from medical imaging. The BiLSTM module is used strictly for ordered-input representation learning rather than longitudinal disease-progression modelling. For RSNA mammography, the ordered inputs are complementary CC and MLO views; for LIDC-IDRI, they are neighbouring CT slices around a target nodule. Multimodal fusion is not applied uniformly across both datasets: cross-attention-based image–metadata fusion is evaluated exclusively for the RSNA mammography task using leakage-safe patient age and implant status, whereas the primary LIDC-IDRI analysis remains image-only. This dataset-specific formulation prevents the multimodal capability from being overstated and reflects the information genuinely available in each public benchmark.

The main contributions of this study are as follows. First, it introduces a unified framework that combines CNN feature extraction, lesion-aware graph construction, GAT-based structural modelling, and BiLSTM-based ordered-input encoding within one diagnostic pipeline. Second, it defines an explicit leakage-control protocol for multimodal learning. In the RSNA mammography experiment, only patient age and implant status are used as metadata inputs, whereas biopsy status, invasive status, cancer labels, and other post-diagnostic variables are excluded. In the LIDC-IDRI experiment, the primary analysis is image-only because malignancy scores and radiologist semantic ratings are annotation-derived variables rather than independent pre-diagnostic metadata. Third, this study distinguishes multi-view or slice-sequence modelling from true longitudinal progression modelling and avoids claims of temporal disease evolution that are unsupported by the available datasets. Fourth, the experimental design explicitly separates the RSNA multimodal experiment from the image-only LIDC-IDRI experiment and applies a leakage-control protocol. Multimodal fusion is evaluated only for the RSNA mammography task using patient age and implant status, which are available before the diagnostic outcome. Biopsy status, invasive status, cancer labels and other outcome-derived variables are excluded from the input representation. LIDC-IDRI is evaluated as an imaging-only task in the primary analysis because the available nodule semantic attributes are radiologist-derived annotations rather than independent clinical metadata. Fifth, the interpretability analysis is linked to case-level model evidence rather than presented only as a general visual explanation.

Recent cancer imaging studies have moved beyond conventional CNN classification toward transformer-based diagnostic models, graph-based medical image analysis, multimodal fusion and medical foundation models. Swin Transformer and UNETR-style architectures improve global contextual modelling but often require large data volumes and do not explicitly encode lesion-centred structural priors [11,12,13]. Graph-based medical imaging studies improve relational reasoning between anatomical regions, although many remain image-only and do not incorporate ordered image representations or clinical metadata [14,15,16]. Multimodal learning studies have shown that combining imaging data with electronic health records or clinical descriptors can improve diagnostic context, but simple concatenation remains limited because it does not model patient-specific cross-modal interactions [17,18,19,20]. Recent work on medical vision–language models, teacher–student knowledge distillation and personalised federated learning further demonstrates that medical AI is shifting toward richer multimodal, efficient and privacy-aware systems [21,22,23]. Recent advances in multimodal medical AI motivate the integration of complementary imaging and clinical information within a unified diagnostic framework. In the proposed architecture, standard transformer-based cross-attention is adopted as a feature fusion mechanism to integrate leakage-safe clinical metadata with image representations. The methodological contribution of this work is not the attention formulation itself, but its integration with graph-aware relational learning, sequence-aware modelling and leakage-controlled multimodal fusion for medical image analysis.

Overall, this work aims to advance medical image analysis by developing a context-aware and interpretable framework for cancer detection and sequence-aware risk estimation. The framework is not presented as a validated longitudinal progression model; rather, it is designed to exploit ordered mammographic views and volumetric CT slice continuity where true follow-up data are unavailable. Recent medical AI research has expanded from conventional CNN classifiers toward transformer-based architectures, graph-based reasoning, multimodal fusion, vision–language modelling, knowledge distillation, and privacy-aware federated learning [24,25]. These frontier directions are relevant to the present work for three reasons. First, they show that modern diagnostic systems increasingly benefit from combining complementary sources of information rather than relying on isolated image features. Second, they emphasise the need for computationally efficient models that remain deployable in realistic clinical environments rather than depending exclusively on very large foundation-scale architectures. Third, they highlight the importance of interpretability, robustness, and privacy-aware design when moving from benchmark performance toward practical medical AI deployment. The framework proposed in this study is positioned within this landscape as a compact architecture that integrates CNN-based spatial encoding, lesion-aware graph construction, GAT-based structural reasoning, BiLSTM-based ordered-input modelling, and leakage-controlled metadata fusion. It does not aim to replace foundation models or claim a novel attention formulation; rather, it evaluates whether these complementary components can be combined in a clinically constrained and interpretable diagnostic pipeline.

## 2. Related Work

Research in medical image analysis for cancer detection has evolved significantly over the past decade, driven largely by advances in machine learning and, more recently, deep learning [26,27,28]. This research can be categorised into several interconnected domains: convolutional neural networks for image-based diagnosis, attention-based learning for feature refinement, graph-based approaches for relational modelling, temporal learning for ordered-view modelling, and multimodal frameworks integrating heterogeneous data sources. While each of these research directions has demonstrated promising results independently, there remains a clear gap in unified frameworks that combine all these capabilities for comprehensive cancer detection and progression analysis.

### 2.1. Convolutional Neural Networks in Medical Imaging

CNNs have become the foundation of most modern medical imaging systems due to their ability to automatically learn hierarchical representations from raw data [3]. Early work by Litjens et al. [1] provided a comprehensive survey of deep learning applications in medical imaging, demonstrating that CNN-based models significantly outperform traditional machine learning approaches in tasks such as tumour detection and segmentation. Similarly, Esteva et al. [2] showed that deep CNNs could achieve dermatologist-level performance in skin cancer classification, highlighting the transformative potential of deep learning in clinical diagnostics.

Subsequent research introduced advanced architectures such as ResNet, DenseNet, and EfficientNet, which improved feature extraction through deeper networks and optimised parameter efficiency. For example, He et al. [11] proposed residual learning, enabling the training of very deep networks without degradation, while Tan and Le [12] introduced EfficientNet, which systematically scales network depth, width, and resolution to achieve improved performance with fewer parameters.

Despite their success, CNN-based approaches exhibit inherent limitations. These models operate on Euclidean grid structures and primarily focus on local receptive fields, making it difficult to capture long-range dependencies and complex spatial relationships between tumour regions. In medical imaging, where tumour morphology and its interaction with surrounding tissues are crucial diagnostic indicators, this limitation restricts the interpretability and clinical relevance of CNN-based models.

### 2.2. Attention Mechanisms for Feature Enhancement

To address the limitations of standard CNNs, attention mechanisms have been widely incorporated into deep learning models. Attention allows networks to dynamically focus on the most relevant parts of an input, thereby improving feature representation and classification performance [3]. Liu et al. [14] introduced attention-based reasoning models, demonstrating improved performance in tasks requiring contextual understanding. Crasta et al. [27] further explored attention mechanisms in deep learning, showing their effectiveness in enhancing interpretability and classification accuracy.

In earlier work, attention mechanisms were integrated with recurrent neural networks to improve feature weighting in sequential data. For instance, attention-enhanced LSTM models have been shown to improve classification performance by emphasising important features within sequences. Liz-López et al. [28] highlighted that attention mechanisms mimic human cognitive processes by selectively focusing on relevant information, making them particularly suitable for complex tasks such as medical diagnosis.

In medical imaging, attention-based CNN models have been used to highlight tumour regions and improve segmentation accuracy. However, most attention-based approaches still operate within a grid-based representation and do not explicitly model relationships between different regions of an image. As a result, while attention improves feature importance, it does not fully address the need for structural understanding.

### 2.3. Graph Neural Networks for Structural Modelling

GNNs have emerged as a powerful alternative for modelling non-Euclidean data structures. Unlike CNNs, which operate on regular grids, GNNs can represent data as graphs, where nodes correspond to entities (e.g., image patches or anatomical regions) and edges represent relationships between them [6]. Kipf and Welling [5] introduced GCNs, which extend convolution operations to graph structures, enabling the learning of node representations based on their neighbourhood.

Velickovic et al. [6] further advanced this concept with GATs, which incorporate attention mechanisms to dynamically weight neighbouring nodes. This approach is particularly useful in medical imaging, where the importance of neighbouring regions may vary depending on context. For example, tumour boundaries and adjacent tissues may have different levels of diagnostic significance, which can be captured effectively through graph attention.

Recent studies have applied GNNs to medical imaging tasks such as brain tumour segmentation and lung nodule classification. These approaches demonstrate improved performance by modelling spatial dependencies and relationships between regions. However, most existing GNN-based methods focus solely on spatial modelling and do not incorporate temporal or multimodal information. This limits their applicability in real-world clinical scenarios, where ordered-view modelling and patient-specific context are critical.

### 2.4. Sequence-Aware Modelling in Medical Imaging

Temporal modelling plays a crucial role in understanding ordered-view modelling, particularly in cancer diagnosis where changes over time can provide important diagnostic cues. RNNs, including LSTM networks, have been widely used for sequential data analysis due to their ability to capture long-term dependencies. Hochreiter and Schmidhuber [7] introduced LSTM networks to address the vanishing gradient problem in standard RNNs, enabling effective learning of temporal patterns.

Graves and Schmidhuber [8] extended this work by proposing BiLSTM, which processes sequences in both forward and backward directions, providing richer contextual information. These models have been successfully applied in areas such as speech recognition and natural language processing, and more recently in medical imaging for analysing sequential scans.

Transformer-based architecture has further improved sequence-aware modelling through self-attention mechanisms. Vaswani et al. [9] introduced the transformer model, which eliminates recurrence and relies entirely on attention mechanisms to model dependencies. This approach has shown superior performance in various sequence modelling tasks and is increasingly being explored in medical imaging.

In this study, sequence-aware modelling is interpreted conservatively. The BiLSTM module learns ordered-view and slice-sequence dependencies, not longitudinal disease evolution. This design is still meaningful because adjacent CT slices and paired mammographic views contain complementary spatial-context information that may be lost when each image is classified independently. The limitation is that external longitudinal datasets are needed before claims about disease progression can be made.

### 2.5. Multimodal Learning in Healthcare

Clinical decision-making often relies on multiple sources of information, including imaging data, patient demographics, medical history, and laboratory results. Multimodal learning aims to integrate these heterogeneous data sources to improve predictive performance and robustness. Baltrusaitis et al. [10] provided a comprehensive survey of multimodal machine learning, highlighting the importance of combining complementary information from different modalities.

Traditional multimodal approaches use simple feature concatenation or early fusion techniques, which may not effectively capture interactions between modalities. Recent work has introduced attention-based fusion mechanisms, such as cross-attention, which allow models to dynamically learn relationships between different data sources. These methods have shown improved performance in tasks such as disease diagnosis and prognosis prediction.

In cancer detection, multimodal frameworks have been used to combine imaging data with genomic and clinical information. While these approaches demonstrate improved accuracy, they often suffer from challenges such as data alignment, missing modalities, and increased model complexity. Furthermore, most multimodal models do not incorporate graph-based or temporal learning, limiting their ability to fully exploit the available data.

### 2.6. Hybrid Deep Learning Architectures

Hybrid architectures that combine multiple deep learning components have gained increasing attention in recent years. For example, CNN-RNN models integrate spatial feature extraction with sequence-aware modelling, providing a more comprehensive representation of data. Li et al. [29] demonstrated that combining CNN and RNN architectures improves classification performance by leveraging both spatial and sequential information.

Similarly, attention-based hybrid models have been used to enhance feature representation and improve interpretability [30]. These models combine convolutional layers with attention mechanisms to focus on relevant regions while capturing hierarchical features. However, most hybrid models are limited to two components (e.g., CNN + RNN or CNN + attention) and do not integrate graph-based learning or multimodal fusion.

#### Emerging Medical AI: Vision–Language Models, Knowledge Distillation and Federated Learning

Recent medical AI research has moved beyond task-specific image classification toward broader multimodal and deployable learning paradigms. Vision–language models connect medical images with reports, clinical notes, and semantic medical knowledge, enabling richer diagnostic reasoning than conventional image-only systems. Their main relevance to the present study is conceptual where they demonstrate that multimodal learning is most useful when each information source is clearly defined and reflects realistic clinical availability rather than arbitrary feature aggregation.

Knowledge distillation and teacher–student learning address a different but equally important challenge: how to retain strong predictive performance while reducing computational burden. This direction is relevant because medical imaging models often face deployment constraints related to memory, latency, and hardware availability. The present framework is more compact than large multimodal foundation models, and its modular design is consistent with the broader goal of balancing representational richness with practical feasibility.

Personalised federated learning further highlights the importance of privacy-aware model development for multi-institutional medical imaging. Such methods aim to preserve institutional data locality while adapting models to heterogeneous patient populations and imaging protocols [31]. Although federated training is outside the scope of this study, it provides a useful future deployment perspective. In this context, the current work is best positioned not as a frontier foundation model, but as an interpretable and computationally manageable architecture that can serve as a building block for future multimodal, privacy-aware, and clinically deployable diagnostic systems.

### 2.7. Research Gap and Motivation

A review of the existing literature reveals five specific limitations that motivate the proposed framework: (1) most CNN models learn local grid-based features but do not explicitly represent relationships between suspicious regions; (2) many medical imaging studies use static image classification and do not exploit ordered views, slices or patient-level sequences; (3) clinical metadata is often ignored or added through simple concatenation rather than attention-guided fusion; (4) existing hybrid models usually combine only two components such as CNN-LSTM or CNN-attention, leaving graph, sequence and multimodal learning fragmented; and (5) interpretability is often post hoc rather than connected to graph attention, temporal contribution and case-level prediction evidence.

- Lack of structural modelling: CNN-based models do not explicitly capture relationships between tumour regions.
- Limited temporal analysis: Most studies treat imaging data as static and ignore ordered-view modelling.
- Insufficient multimodal integration: Many models fail to effectively combine imaging and clinical data.
- The reviewed studies show that CNN-based models are effective for extracting local imaging features, but they are limited in modelling long-range structural relationships between suspicious regions. Transformer-based models improve global dependency learning, although they often require large datasets and may not explicitly preserve lesion-centred medical structure. Graph-based approaches are useful for representing relationships among anatomical or lesion regions, but many do not incorporate ordered imaging inputs or clinical metadata. Multimodal approaches improve diagnostic context by integrating imaging and non-imaging variables, but many rely on simple concatenation and do not model adaptive cross-modal interactions. The proposed framework is positioned at the intersection of these limitations by combining CNN feature extraction, lesion-aware graph construction, GAT-based relational modelling, BiLSTM-based sequence-aware representation learning and cross-attention metadata fusion within a single diagnostic pipeline. Table 1 shows the strengths, limitations and connection of reviewed approaches to the proposed framework.

While attention mechanisms have improved feature weighting and classification performance, as demonstrated in prior work, there is a need to extend these concepts to more complex representations involving spatial relationships and ordered-input dependencies.

## 3. The Proposed Cancer Detection and Sequence-Aware Classification Architecture

**Figure 1:**
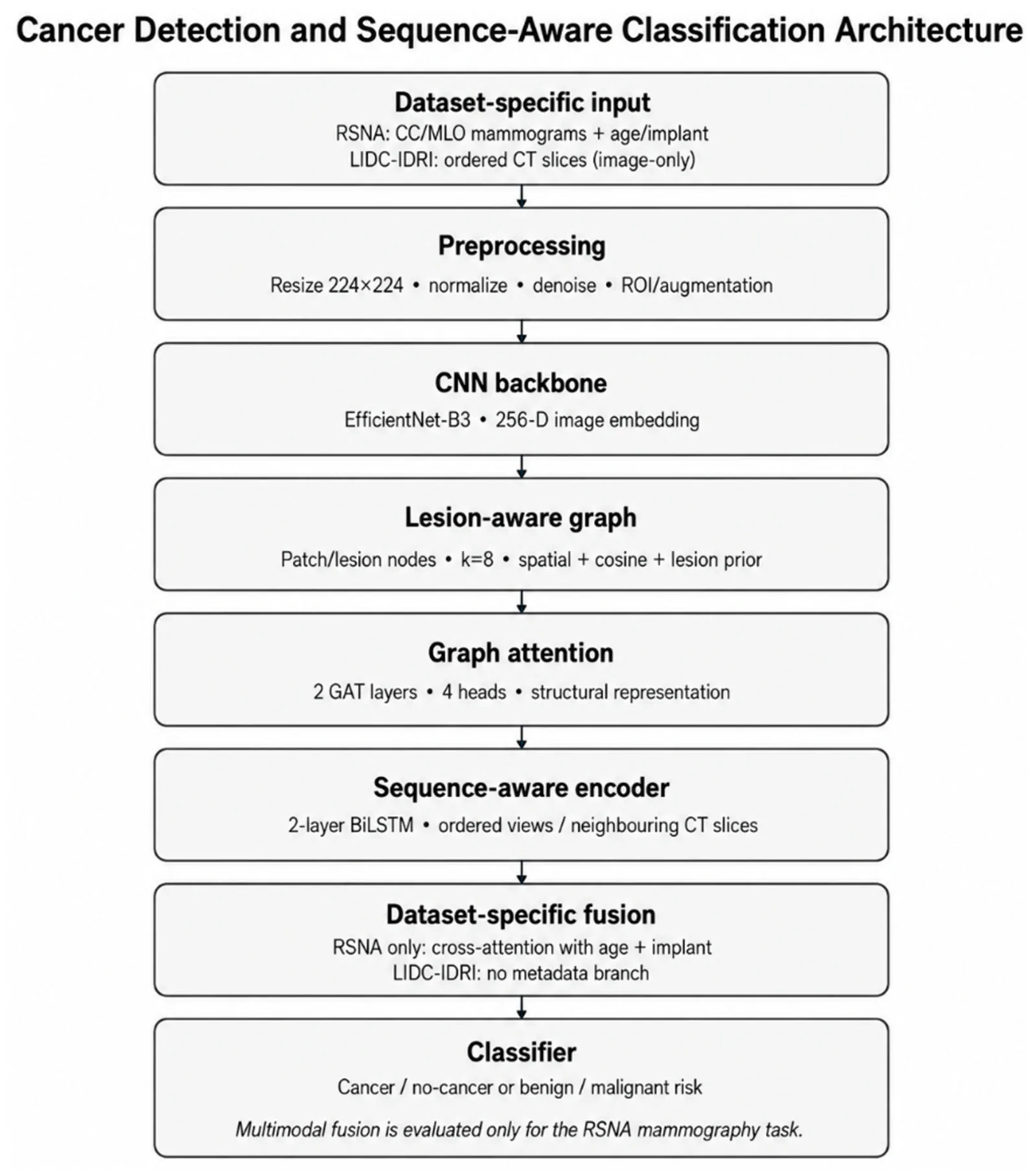
Cancer detection and sequence-aware classification architecture with dataset-specific RSNA multimodal fusion.

The proposed framework is designed to analyse medical imaging data through four coordinated stages: spatial feature extraction, graph-based structural modelling, ordered-input sequence encoding, and classification. A leakage-controlled multimodal fusion branch is used only in the RSNA mammography experiment, where limited pre-diagnostic metadata are available. The primary LIDC-IDRI experiment remains image-only. This dataset-specific design is central to the methodological validity of this study because it prevents annotation-derived or post-diagnostic variables from being treated as clinical predictors, as shown in Figure 1.

The first stage receives medical images from mammography or CT datasets. These images are resized, normalised, denoised and augmented to improve model generalisation. The second stage uses a CNN backbone such as EfficientNet, ResNet, DenseNet, ConvNeXt or Swin Transformer to extract high-level spatial features. The third stage converts extracted feature maps into graph structures, where nodes represent patches or lesion-centred regions and edges represent spatial and feature-based relationships. The fourth stage applies GAT to assign adaptive importance to neighbouring nodes and highlight clinically relevant region-to-region interactions. The fifth stage applies a sequence encoder to ordered mammography views or CT slices. This sequence-aware component is not described as true longitudinal progression unless repeated scans from the same patient at different time points are available.

The third stage converts the extracted feature maps into a graph structure. Each image patch or region of interest is treated as a node, while edges are created based on spatial closeness and feature similarity. This allows the model to understand how suspicious regions relate to surrounding tissue rather than treating each region independently. The fourth stage applies GAT. GAT assigns attention weights to neighbouring nodes, allowing the model to focus more strongly on clinically important regions. This improves the detection of irregular tumour margins and subtle tissue changes.

The fifth stage uses a sequence-aware encoder, implemented as BiLSTM, to capture dependencies across ordered mammography views or neighbouring CT slices. It is not used to claim true longitudinal disease progression in RSNA or LIDC-IDRI because these datasets do not provide repeated patient follow-up scans with progression labels. Instead, the sequence encoder learns contextual relationships among ordered image-derived graph representations.

The input modalities used in this study are limited to mammography and CT imaging. MRI is not used in experimental evaluation and should therefore not be shown as an input modality in the architecture figure. The architecture is designed to be extensible to MRI in future work, but the present implementation and reported results are based only on RSNA mammography and LIDC-IDRI CT data.

Finally, the fused representation is passed through fully connected layers and a Softmax or sigmoid classifier. The model produces binary cancer detection output and benign/malignant risk estimation. Sequence-aware risk output is interpreted as contextual risk stratification from ordered imaging inputs, not as confirmed disease progression.

### 3.1. Dataset-Specific Inputs and Leakage-Control Protocol

Innovation 1: Graph Construction Strategy for Structural Tumour Representation

A key innovation of the proposed framework lies in the graph construction strategy used to transform conventional image feature maps into graph-based representations. After image preprocessing and feature extraction using the CNN backbone, the input medical image (X) is transformed into a high-dimensional feature map (F).

The resulting feature map is partitioned into non-overlapping 16 × 16 patches. Each patch represents a local anatomical region and is converted into a graph node. Let the graph be shown as:

(1) $$X\;\in \;R^{H\times W\times C}$$

(2) $$G=(V,\;E),\;V=\{v_{1},v_{2},\ldots ,\;v_{N}\}$$

where (V) denotes the set of nodes and (E) denotes the set of edges connecting related regions.

Each node (*v_i_*) is associated with a feature embedding (*h_i_*) extracted from the corresponding image patch. To capture structural dependencies, edges are established using a hybrid similarity criterion that combines visual similarity, spatial proximity and lesion-prior constraints. Cosine similarity between node embeddings *h_i_* and *h_j_* is calculated as:

(3) $$S_{ij}=\;\frac{h_{i}^{T}h_{j}}{\left\|h_{i}\right\|\left\|h_{j}\right\|}$$

The edge weight between two candidate nodes is defined as:

(4) $$W_{ij}=\;\lambda_{1}S_{ij}+\lambda_{2}P_{ij}+\lambda_{3}M_{ij}$$

where *S_ij_* is cosine feature similarity, *P_ij_* is normalised spatial proximity, *M_ij_* is the lesion-prior constraint, and *λ*_1_, *λ*_2_ and *λ*_3_ control the relative contribution of each term.

The weighted adjacency matrix is then constructed as: *A_ij_* = *w_ij_* if *j* ∈ *N_k_* (i) or *M_ij_* > τ; otherwise *A_ij_* = 0.

(5) $$F=f_{CNN}\left(X\right),F\in R^{h\times w\times d}$$

This graph formulation goes beyond a generic patch graph because suspicious lesion regions receive higher structural priority. It preserves local tissue continuity, feature-level similarity and clinically meaningful lesion relationships, thereby improving the identification of tumour heterogeneity and subtle pathological patterns.

The edge weight between two connected nodes is defined as:

(6) $$w_{ij}=\alpha S_{ij}+(1-\alpha )D_{ij}$$

where *D_ij_* represents the normalised spatial distance and α controls the balance between feature similarity and spatial proximity.

These weights form the weighted adjacency matrix (*A*):

(7) $$A={\left[w_{ij}\right]}_{NxN}$$

which captures both local and global contextual relationships among tumour regions and surrounding tissues. This graph representation preserves structural information that is often lost in conventional convolutional architectures, thereby improving the identification of tumour heterogeneity and subtle pathological patterns.

Innovation 2: Hybrid Graph–Sequence Interaction Mechanism

The second innovation is the integration of graph-based representation learning with sequence-aware modelling through a hybrid graph–sequence architecture. In the proposed framework, the GAT is applied prior to the BiLSTM module. The GAT first aggregates structural information from neighbouring nodes using an attention mechanism. The attention coefficient between nodes (*i*) and (*j*) is calculated as:

(8) $$Attention\;(Q,\;K,\;V)=softmax\left(\frac{QK^{T}}{\sqrt{d_{k}}}\right)V$$

(9) $$\alpha_{ij}=\frac{\exp(LeakyReLU\;(a^{T}[Wh_{i}\left\|Wh_{j}]))\right.}{\sum_{k\in N(i)}\exp(LeakyReLU\;(a^{T}[Wh_{i}\left\|Wh_{k}]))\right.}$$

The node representation is then updated as:

(10) $$h_{i}^{\prime }=\sigma \left(\sum_{j\epsilon N(i)}\alpha_{ij}Wh_{j}\right)$$

where (W) denotes a learnable weight matrix and (σ) represents a nonlinear activation function. The resulting graph-level representation (Z_t_) is subsequently passed to the BiLSTM module to model ordered imaging sequences:

(11) $$H_{t}=BiLSTM(Z_{t})$$

Applying GAT before BiLSTM ensures that sequence modelling operates on structurally enriched tumour representations rather than isolated CNN features. This information flow allows the framework to jointly capture spatial dependencies and sequence-aware relationships, producing more informative embeddings for cancer diagnosis.

Innovation 3: Leakage-Safe Cross-Attention Metadata Fusion

The proposed framework adopts the standard scaled dot-product cross-attention mechanism introduced in the transformer architecture to enable adaptive fusion between imaging representations and leakage-safe clinical metadata. Rather than proposing a new attention formulation, this study focuses on evaluating how cross-attention can be incorporated within a graph-aware and sequence-aware medical imaging framework while preventing information leakage from outcome-related variables. For the RSNA mammography experiment, the metadata vector contains only patient age and implant status. Patient age is represented as a continuous variable and standardised using statistics calculated from the training set. Implant status is represented as a binary variable. Mammographic laterality and view are used to group and order the image inputs but are not treated as clinical metadata in the fusion vector.

The following RSNA variables are explicitly excluded from the metadata input: biopsy status, invasive status, cancer outcome, difficult-negative-case indicators and any variable generated after diagnostic assessment. These fields may contain direct or indirect information about the reference label and would therefore create target leakage if included as predictors.

For LIDC-IDRI, the primary experiment does not use a separate metadata branch. The radiologist-assigned malignancy score is used only to derive the target class and is never supplied to the model as an input. Semantic ratings such as subtlety, margin, lobulation, spiculation, texture, sphericity, calcification and internal structure are also excluded from the primary multimodal analysis. Although these annotations are clinically meaningful, they are derived from radiologist interpretation of the same CT images and are not independent pre-diagnostic patient metadata. Including them could make the comparison with image-only models clinically unrealistic.

Let (*H_I_*) denote the sequence-aware imaging representation and let (*H_C_*) denote the encoded RSNA metadata representation. Imaging features are projected to query vectors, while the metadata representation is projected to key and value vectors. The mathematical formulation of the cross-attention module follows the conventional transformer attention mechanism. The equations presented below are included for completeness and reproducibility of the implementation rather than as a methodological contribution. The novelty of the proposed framework lies in the integration strategy, leakage-control protocol and interaction between graph-based, sequence-aware and multimodal learning components.

(12) $$Q=H_{I}W_{Q},\;\;\;\;\;K=H_{C}W_{K},\;\;\;\;\;\;\;V=H_{C}W_{V}$$

The metadata-conditioned imaging representation is calculated using standard scaled dot-product cross-attention as described in (7). The final fused representation is obtained by combining the original imaging representation with the attended metadata representation:

(13) $$H_{F}=[H_{I}\left\|H_{A}\right.H_{I}]$$

Unlike existing multimodal medical imaging approaches that typically concatenate imaging and metadata features directly, the proposed framework uses standard cross-attention to enable adaptive interaction between the image representation and leakage-safe metadata. The contribution therefore lies in the design of the overall diagnostic pipeline rather than in introducing a new attention formulation. The proposed architecture combines lesion-aware graph construction, graph attention, sequence-aware encoding and adaptive multimodal fusion within a single end-to-end framework.

Innovation 4: Unified Graph-Aware and Sequence-Aware Multimodal Architecture

The final innovation is the development of a unified architecture that simultaneously integrates CNN-based feature extraction, graph representation learning, sequence-aware modelling, and multimodal fusion within a single end-to-end framework. Existing approaches such as CNN-LSTM, CNN-GAT, and CNN-Metadata fusion capture only partial information from medical data. In contrast, the proposed framework learns a comprehensive representation:

(14) $$H_{final}=Fusion\left(BiLSTM\;\left(GAT\left(CNN\;\left(X\right)\right)\right),\;C\right)$$

where (*X*) represents the input medical image and (*C*) denotes clinical metadata. The final prediction is generated through a fully connected classification layer:

The final classification equation is given as:

(15) $$\overset{\wedge }{y}=Softmax\;({WH}_{final}+b)$$

This unified formulation enables simultaneous modelling of spatial image features, structural tumour relationships, sequence-aware dependencies, and patient-specific clinical information. Consequently, the framework can learn complementary information from multiple sources, leading to more accurate, interpretable, and clinically meaningful cancer detection compared with conventional CNN-LSTM, CNN-GAT, or CNN-Metadata architectures.

Although the fusion module uses the standard transformer cross-attention mechanism, its integration with graph-based relational modelling, sequence-aware representation learning and leakage-controlled metadata selection constitutes the principal architectural contribution of the proposed framework.

The datasets used in this study are selected from publicly available Kaggle repositories to ensure reproducibility and relevance to real-world clinical applications. The primary dataset employed is the RSNA Breast Cancer Detection dataset, which can be accessed at https://www.kaggle.com/competitions/rsna-breast-cancer-detection (accessed on 21 June 2026). This dataset provides high-resolution mammography images along with patient-level metadata, making it suitable for spatial feature extraction, graph-based modelling, and multimodal fusion. To further validate the robustness and generalisability of the proposed framework across different imaging modalities, the LIDC-IDRI lung CT dataset is also utilised, available at https://www.cancerimagingarchive.net/collection/lidc-idri/ (accessed on 21 June 2026). This dataset includes volumetric CT scans with annotated nodules, which supports structural learning and natural sequence-based sequence-aware modelling. The combination of these datasets ensures that the proposed approach is evaluated on both 2D and 3D imaging data while incorporating clinical context where available.

Overall, the selected datasets satisfy the key requirements of the proposed methodology: they provide high-quality imaging data for spatial feature extraction, structured or sequential information for sequence-aware modelling, and associated metadata for multimodal integration. By meeting these criteria, the datasets enable a comprehensive evaluation of the framework’s ability to jointly model spatial dependencies, ordered-input dependencies, and clinical context, which are critical for accurate early cancer detection and progression analysis.

### 3.2. Overall Architecture

**Figure 2:**
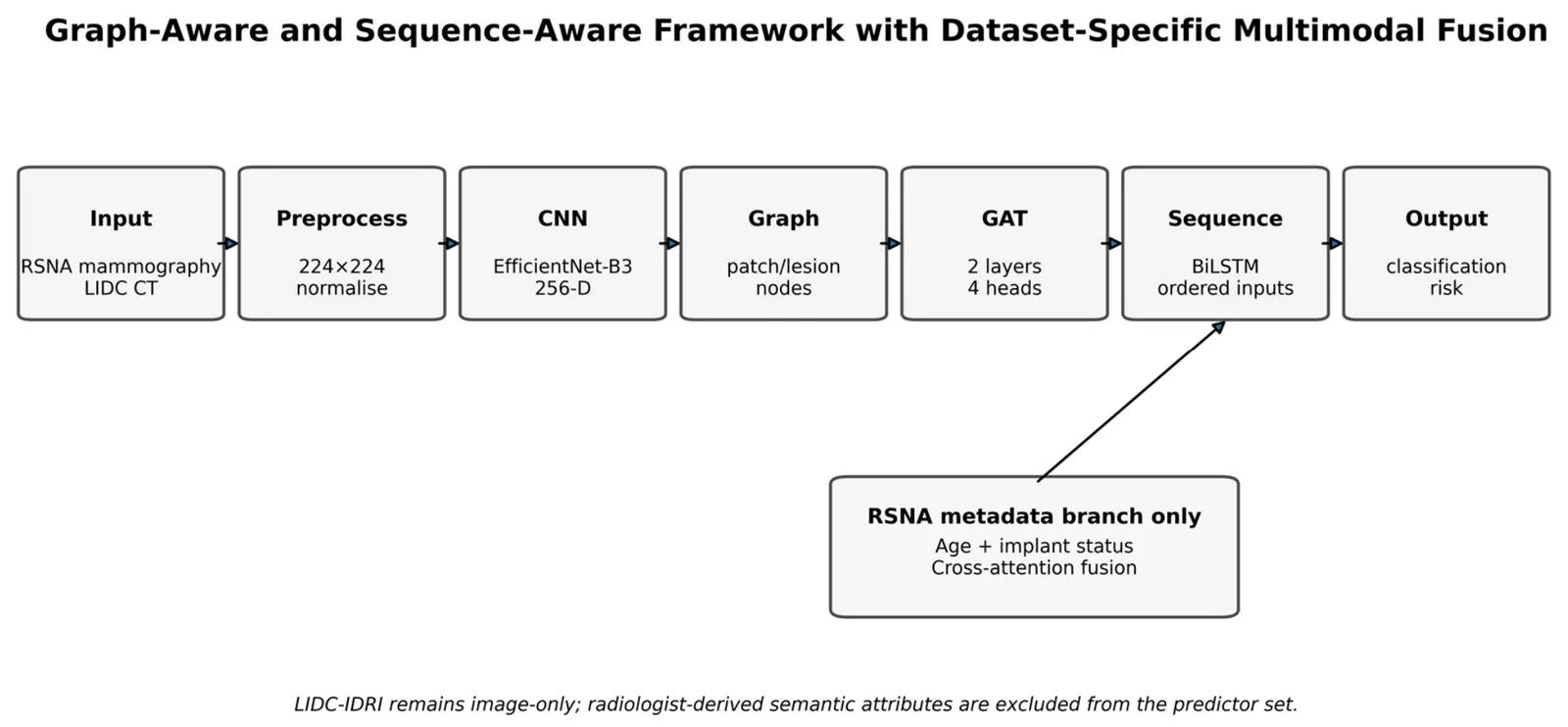
Overall graph-aware and sequence-aware framework. Cross-attention metadata fusion is evaluated only for RSNA mammography; LIDC-IDRI remains image-only.

The architecture follows a sequential pipeline where imaging data is transformed into spatial, structural, and sequence-aware representations before final classification. Figure 2 shows the overall architecture of the proposed graph-aware and sequence-aware multimodal deep learning framework.

The overall architecture of the proposed framework is designed as a sequential pipeline where each stage transforms the data into a richer and more meaningful representation for cancer detection and progression analysis. Each step in the architecture plays a specific role in addressing limitations of traditional models by incorporating spatial, structural, temporal, and multimodal learning. Repetitive descriptions in the architecture discussion were consolidated to improve clarity and readability.

#### 3.2.1. Dataset-Specific Inputs

The framework receives mammography images from the RSNA dataset and thoracic CT images from LIDC-IDRI. MRI is not included in the experimental evaluation. For RSNA, the imaging input consists of available CC and MLO mammographic views grouped at the patient and breast level. Patient age and implant status form the only non-image metadata inputs used in the multimodal experiment. Laterality and mammographic view are used to organise the image inputs but are not included as clinical predictors. For LIDC-IDRI, the model receives ordered CT slices surrounding the annotated nodule. The primary LIDC-IDRI experiment is image-only. Radiologist malignancy scores are used to establish the classification target and are not passed into the model. Other radiologist-assigned semantic ratings are also excluded from the metadata branch because they are derived from interpretation of the same imaging examination.

This dataset-specific definition prevents diagnostic outcome information from entering the predictor set and ensures that the multimodal experiment represents information that could reasonably be available when an image is assessed.

#### 3.2.2. Preprocessing and Normalisation

Once the data is loaded, it undergoes preprocessing to ensure consistency and quality across all samples. Medical images often vary in size, contrast, and acquisition conditions, which can negatively affect model performance if not standardised. In this stage, images are resized to a fixed resolution suitable for the CNN backbone, and intensity values are normalised to a common scale. Noise removal techniques are applied to reduce artefacts, while data augmentation methods such as rotation, flipping, and contrast adjustment are used to improve generalisation and prevent overfitting. Additionally, region-of-interest (ROI) extraction may be performed to focus the model on relevant anatomical areas where tumours are likely to appear. This step ensures that the input data is clean, standardised, and informative before feature extraction.

#### 3.2.3. CNN Feature Extraction (EfficientNet/ResNet)

After preprocessing, the images are passed through a convolutional neural network, which acts as the backbone of the architecture. Models such as EfficientNet or ResNet are used due to their ability to extract hierarchical feature representations efficiently. In this stage, CNN learns spatial features such as edges, textures, shapes, and intensity variations that are indicative of tumour presence. These features are captured in the form of high-dimensional feature maps, where each channel represents a different learned pattern. While CNNs are highly effective at capturing local spatial information, they treat the image as a regular grid and do not explicitly model relationships between different regions. This limitation motivates the transition to a graph-based representation in the next stage.

#### 3.2.4. Graph Construction (Nodes = Patches, Edges = Relationships)

The feature maps produced by CNN are transformed into graph structures using an explicit operational procedure. Let the CNN output feature map be divided into non-overlapping or weakly overlapping patch embeddings. Each patch embedding becomes one node in the graph. For mammography, nodes are generated from 2D feature-map patches, with additional emphasis on lesion-suspicious regions when region-of-interest information is available. For CT, nodes are generated from slice-level or nodule-centred 3D neighbourhood patches, depending on the availability of nodule annotations.

Edges are constructed using a hybrid adjacency rule. First, spatial adjacency connects each node to its local neighbouring patches. Second, feature adjacency connects each node to its k most similar nodes using cosine similarity of CNN embeddings. In the revised implementation description, k is treated as a tunable hyperparameter, with k = 8 used as the default setting. Edge weights combine normalised spatial distance and embedding similarity. The resulting graph is patient-specific and dynamically constructed after CNN feature extraction rather than fixed in advance. A global attention pooling layer is then used to aggregate node representations into a graph-level imaging representation.

##### Medical-Prior-Guided Graph Construction

The proposed graph construction is extended beyond simple patch proximity and feature similarity by incorporating lesion-aware medical priors. After CNN feature extraction, each image is divided into patch-level embeddings. However, not all patches are equally informative in medical imaging. Therefore, lesion-suspicious regions are assigned to higher structural priority during graph formation. In mammography, suspicious regions may include dense asymmetrical tissue, mass-like regions, calcification patterns or abnormal texture regions. In CT, nodule-centred regions, slice continuity and radiologist-provided nodule annotations are used to guide node selection.

Let (*h_i_*) and (*h_j_*) denote two node embeddings. The edge weight between nodes (*i*) and (*j*) is defined as in (4). The lesion-prior term increases edge strength between patches located within or near suspicious anatomical regions.

(16) $$A_{ij}=\left\{\begin{array}{ll} w_{ij}, & j\in N_{k}\left(i\right)\;\mathrm{or}\;M_{ij}>\tau \\ 0, & \mathrm{otherwise} \end{array}\right.$$

where *N_k_*(*i*) denotes the k-nearest neighbours of node *i* and *τ* is the lesion-prior threshold. This design preserves local tissue continuity, feature-level similarity and clinically meaningful lesion relationships.

Cross-attention is introduced because simple concatenation treats all imaging and clinical variables as equally important [32]. In clinical practice, however, metadata relevance varies across patients. By using imaging features as queries and clinical metadata as keys and values, the model learns which clinical attributes are most relevant to each image-derived representation. This is particularly important in cancer imaging because similar visual patterns may have different diagnostic implications depending on age, view type, laterality, lesion morphology or nodule annotation [33,34]. Cross-attention therefore provides adaptive multimodal fusion and stronger interpretability than static concatenation.

#### 3.2.5. Graph Attention Network (GAT) Processing

Once the graph is constructed, it is processed using a GAT. It assigns attention weights to the edges between nodes, allowing the model to focus more on important relationships while reducing the influence of irrelevant connections. This mechanism is particularly useful for identifying tumour regions, as it enables the model to highlight areas with abnormal characteristics and their interactions with neighbouring tissue. Unlike traditional graph convolution methods, the attention mechanism dynamically adjusts the importance of each node’s neighbours, making the model more flexible and interpretable. This stage enhances the feature representation by incorporating structural information that cannot be captured by CNNs alone.

#### 3.2.6. Sequence-Aware Learning (BiLSTM)

Following the graph-based learning stage, the model incorporates sequence-aware modelling using a BiLSTM network. In the revised manuscript, this module is described cautiously. For RSNA mammography, the sequence may represent ordered imaging views for the same patient, such as CC and MLO views, and should not be interpreted as chronological progression. For LIDC-IDRI, the sequence may represent ordered CT slices within a scan volume, which captures volumetric continuity rather than disease evolution over time. True longitudinal progression analysis would require repeated scans from the same patient across multiple clinical time points and corresponding progression labels.

To verify that the BiLSTM module learns meaningful ordered-input information, the sequence-aware representation is evaluated through ablation experiments. The full model is compared against a variant without BiLSTM and a variant in which the input order is randomly shuffled. If performance decreases after removing BiLSTM or disrupting the input order, this indicates that the sequence encoder contributes useful information beyond static image-level features. For RSNA, this evaluates whether paired mammographic views provide complementary sequence-aware information. For LIDC-IDRI, this evaluates whether ordered CT slices provide useful volumetric continuity around nodules.

(17) $$Z=\{Z_{1},Z_{2},\ldots ,Z_{T}\}$$

(18) $$\underset{h_{t}}{\to }=\underset{LSTM}{\to }\left(Z_{t},\underset{h_{t-1}}{\to }\right)$$

(19) $$\underset{h_{t}}{\leftarrow }=\underset{LSTM}{\leftarrow }\left(Z_{t},\underset{h_{t+1}}{\leftarrow }\right)$$

(20) $$H_{t}=\left[\underset{h_{t}}{\to }||\underset{h_{t}}{\leftarrow }\right]\;$$

The BiLSTM module is employed to capture dependencies across ordered image representations rather than true longitudinal disease progression. For the RSNA mammography dataset, the sequence consists of complementary mammographic views (CC and MLO), while for the LIDC-IDRI dataset the sequence consists of ordered CT slices surrounding the target nodule. Therefore, the BiLSTM is designed to model contextual relationships among ordered imaging features and volumetric continuity rather than temporal progression in the clinical sense. The resulting sequence-aware representation is passed to the metadata-fusion module for the RSNA experiment or directly to the classifier for the image-only LIDC-IDRI experiment.

#### 3.2.7. Leakage-Safe Metadata Definition and Cross-Attention Fusion

The multimodal branch is applied only to the RSNA mammography experiment. The final predictor set is fixed before model training and contains two metadata variables: patient age and implant status. No variable is included conditionally using expressions such as “when available” or “may include”.

Patient age is treated as a continuous variable. Missing age values are imputed using the median calculated from the training subset, after which age is standardised using the training-set mean and standard deviation. Implant status is treated as a binary categorical variable. Missing implant values, if present, are assigned an explicit unknown category rather than inferred from the cancer outcome.

Laterality and mammographic view are used to group the mammograms and determine the ordering of CC and MLO image representations. They are not included in the metadata vector because they describe image acquisition and organisation rather than independent clinical risk factors.

The following variables are excluded from all model inputs:

1. Cancer or malignancy outcome labels;
2. Biopsy status;
3. Invasive cancer status;
4. Difficult-negative-case indicators;
5. Pathology or follow-up outcomes;
6. Any field created after diagnostic assessment;
7. Any variable derived directly from the target label.

For LIDC-IDRI, no separate clinical metadata vector is used in the primary experiment. The radiologist malignancy score is used only for target definition. Subtlety, internal structure, calcification, sphericity, margin, lobulation, spiculation and texture are not provided to the classifier because they are radiologist-derived interpretations of the same CT images. The LIDC-IDRI branch therefore evaluates the CNN–GAT–BiLSTM imaging architecture without metadata fusion.

For the RSNA multimodal experiment, the BiLSTM imaging representation is used as the query, while the encoded age and implant representations provide the keys and values. A standard scaled dot-product cross-attention module is adopted to enable adaptive interaction between imaging features and leakage-safe metadata. This choice follows established transformer methodology and was selected because it provides an effective and reproducible fusion mechanism without introducing additional architectural complexity. Three RSNA variants are evaluated to isolate the contribution of metadata and the projections and fusion are calculated by using (7), (11) and (12).

These equations implement standard scaled dot-product cross-attention; the methodological contribution is the leakage-safe, dataset-specific integration strategy rather than a new attention formulation.

- Imaging architecture without metadata;
- Imaging architecture with direct metadata concatenation;
- Imaging architecture with cross-attention metadata fusion.

This comparison distinguishes the contribution of the metadata itself from the contribution of the fusion strategy. Table 2 shows the dataset-specific variables and leakage-control decisions.

#### 3.2.8. Classification Output

The proposed framework supports binary cancer detection, benign-versus-malignant classification, and sequence-aware risk representation learning. This study does not claim true longitudinal progression prediction because longitudinal temporal labels are not available in the utilised datasets.

Each patch is treated as a node in a graph: G = (V, E), where V = {*v*_1_, *v*_2_, …, *v_N_*} and each node *v_i_* has an embedding $h_{i}\in R^{d}$. The edge set E connects nodes using k-nearest-neighbour spatial adjacency, feature similarity and lesion-prior constraints.

The weighted adjacency matrix follows Equation (7), and the graph is constructed from the hybrid edge weights defined in Equation (6).

Let the input medical image be:

(21) $$F=f_{CNN}\left(X\right),F\in R^{h\times w\times d}\;\hat{y}=\mathrm{softmax}\left(WH_{f}+b\right)\;L=-\sum_{i=1}^{K}y_{i}\log\left({\hat{y}}_{i}\right)$$

CNNs are effective at capturing local spatial patterns such as texture, edges, and tumour boundaries, which are critical for cancer detection [1,2]. However, they lack the ability to model relationships between distant regions, motivating the integration of graph-based learning.

### 3.3. Graph Construction and Representation

The feature map is partitioned into patch embeddings after CNN extraction. If the CNN feature map has spatial dimensions h × w and feature dimension d, each spatial location or local patch is represented as a d-dimensional node vector. For 2D mammography, node coordinates are defined over the feature-map plane. For CT, node coordinates additionally preserve slice order so that volumetric continuity can be represented before sequence encoding. To learn node relationships, a GAT is applied.

The attention coefficient is computed by normalising pairwise compatibility scores over the neighbours of node *i*.

(22) $$A_{ij}=\exp(-||f_{i}-f_{j}|{|}^{2})$$

**Figure 3:**
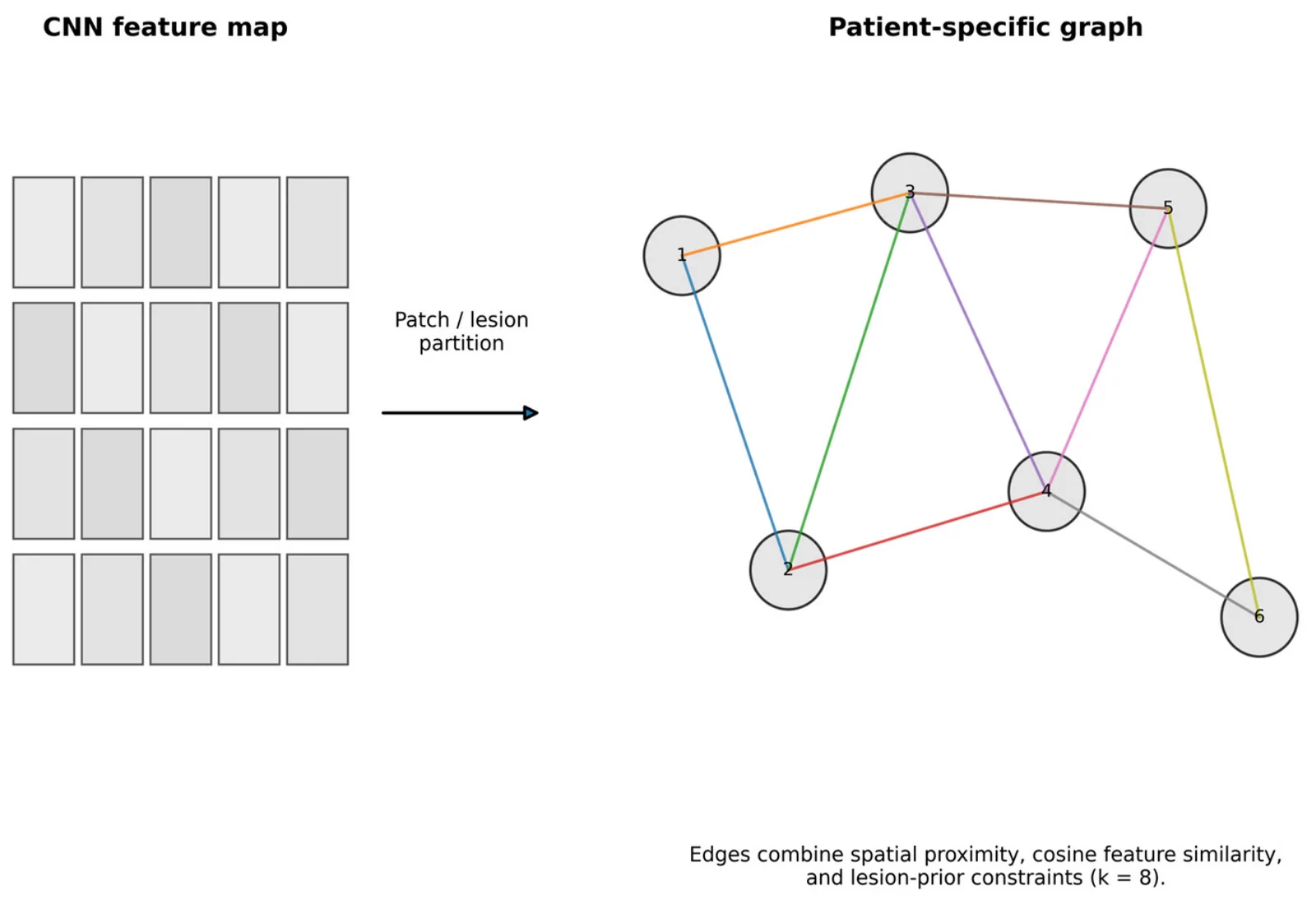
Graph construction from CNN feature maps.

Graph-based representations allow modelling of tumour-region interactions, which are not captured in standard CNN frameworks [5,6]. Figure 3 shows the graph construction from CNN Feature Maps.

### 3.4. Graph Attention Network (GAT)

After graph construction, the patient-specific graph is processed using a GAT to learn the relative importance of neighbouring image regions. Each graph node represents a patch-level or lesion-centred feature embedding, while graph edges represent spatial proximity, feature similarity and lesion-prior relationships.

For each connected pair of nodes *i* and *j*, an unnormalised compatibility score is computed as:

(23) $$e_{ij}=LeakyReLU\left(a^{T}\left[Wh_{i}\|Wh_{j}\right]\right)$$

where *h_i_* and *h_j_* are input node embeddings, *W* is a learnable projection matrix, *a* is the learnable attention vector, and || denotes concatenation.

The compatibility scores are normalised across the neighbourhood of node i using:

(24) $$\alpha_{ij}=\frac{exp(e_{ij})}{\sum_{k\in N(i)}exp(e_{ik})}$$

where $N\left(i\right)$ denotes the neighbouring nodes connected to node i. The coefficient $\alpha_{ij}$ represents the learned importance of node j when updating node i.

The updated representation of node i is calculated by using (9). After the graph attention layers, the updated node representations are aggregated using global attention pooling:

(25) $$Z_{t}=\mathrm{Pool}(\{h_{i}^{\prime }{\}}_{i=1}^{N})$$

The resulting graph-level representation $Z_{t\;}$ summarises the structural relationships among lesion regions and surrounding tissue for the corresponding mammographic view or CT slice. This representation is subsequently supplied to the sequence-aware BiLSTM module described in Section 3.2.6. The GAT module therefore performs structural representation learning only and is not responsible for sequence modelling or multimodal metadata fusion.

### 3.5. Classification Layer

The final classification is performed using (14). Cross-entropy loss is used for optimisation, and this is calculated based on (20). This enables cancer detection, benign-versus-malignant classification and sequence-aware risk estimation. The output should not be interpreted as longitudinal disease progression unless future datasets provide repeated follow-up scans and progression labels.

### 3.6. Algorithmic Representation

The overall computational procedure of the proposed framework is summarised in Algorithm 1. It integrates CNN-based feature extraction, graph-aware representation learning using GAT, sequence-aware modelling using BiLSTM, and cross-attention-based multimodal fusion to generate cancer diagnosis and progression-risk predictions.
**Algorithm 1** Graph-Aware Sequence-Aware Multimodal Deep Learning         Input: Medical images $X=\left\{X_{1},X_{2},\ldots ,X_{N}\right\}$ and clinical data $C=\left\{c_{1},c_{2},\ldots ,c_{N}\right\}$ Output: Prediction $\hat{y\;}$ (cancer diagnosis and progression risk)        1. For each patient i=1 to N  do        2.          Preprocess image $X_{i}$        (resize, normalise, denoise, augment, ROI extraction)        3.          Extract feature map
$$F_{i}=f_{CNN}\left(X_{i}\right)$$        using CNN backbone        4.          Construct graph $$G_{i}=\left(V_{i},E_{i}\right)$$        from $F_{i}$                   (nodes = patches, edges = proximity + similarity)        5.          Obtain node features
$$H_{i}^{\left(0\right)}$$from patch embeddings        6.          Update node representations using GAT: For l=1 to L do Compute attention coefficients $$\alpha_{ij}^{\left(l\right)}$$Update node features $$H_{i}^{\left(l\right)}=\sigma \left(\sum_{j\in N(i)}\alpha_{ij}^{\left(l\right)}W^{\left(l\right)}H_{j}^{\left(l-1\right)}\right)$$        End For        7.    Obtain graph representation $$Z_{i}$$        (e.g., global pooling over nodes)        8. End For        9. Form ordered imaging sequence $$\left\{Z_{1},Z_{2},\ldots ,Z_{T}\right\}$$        for each patient        10. Compute sequence-aware representation $$H_{t}$$        using BiLSTM        11. Fuse imaging and clinical features using cross-attention to obtain $$H_{f}$$        12. Pass $H_{f}$ through fully connected layers        13. Compute prediction $$\hat{y}=\mathrm{softmax}\left(WH_{f}+b\right)$$        14. Compute loss L        and update model parameters        15. End

Table 3 presents the step-by-step explanation of the proposed hybrid framework.

## 4. Results and Discussion

This paper presents a detailed evaluation of the proposed graph-aware sequence-aware multimodal deep learning framework for cancer detection and contextual risk estimation. The results are analysed using multiple quantitative metrics, comparative experiments, ablation studies, computational profiling and clinical relevance discussion to evaluate the contribution of spatial, structural, ordered-imaging and multimodal learning.

### 4.1. Experimental Setup

Data Availability: The RSNA Breast Cancer Detection dataset is available at https://www.kaggle.com/competitions/rsna-breast-cancer-detection (accessed on 20 June 2026) and the LIDC-IDRI dataset is available at https://www.cancerimagingarchive.net/collection/lidc-idri/ (accessed on 20 June 2026).

The experiments are conducted separately for the RSNA mammography task and the LIDC-IDRI lung CT task. For RSNA, the task is breast cancer classification at patient/image level using mammographic images and available metadata. For LIDC-IDRI, the task is lung nodule malignancy or risk classification using CT slices and annotation-derived labels. The datasets are divided into training, validation and testing sets using patient-wise stratified splitting so that images or slices from the same patient do not appear in more than one split. This is essential to reduce data leakage in medical imaging experiments.

The revised experimental protocol reports the number of patients, number of images/slices, class distribution, positive-to-negative ratio, split proportions, image size input, preprocessing operations and augmentation strategy for each dataset. Images are resized to the selected CNN input resolution, intensity-normalised, denoised where required and augmented using clinically safe transformations such as limited rotation, horizontal flipping where anatomically valid, contrast adjustment and random cropping around the region of interest. All experiments use a fixed random seed, and results are reported over multiple independent runs with mean and standard deviation.

The default implementation uses PyTorch 2.4.1 with GPU acceleration. The final configuration includes batch size 32, Adam optimiser, learning rate 0.0001, dropout 0.5, 50 training epochs, early stopping based on validation AUC and cross-entropy loss for classification. Hardware details, including GPU model, memory, CUDA version and training time, are reported to support reproducibility.

The implementation is carried out using a deep learning framework such as PyTorch or TensorFlow. The model is trained on a GPU-enabled system to handle the computational complexity of CNN, GNN, and BiLSTM components. Table 4 shows the training configuration.

#### Implementation Details and Training Configuration

To improve reproducibility, the implementation details of the proposed framework are explicitly defined. All images were resized to 224 × 224 pixels for 2D mammography inputs and CT slices. Pixel intensities were normalised to the range ([0, 1]), followed by z-score normalisation using dataset-level mean and standard deviation. For RSNA mammography, CC and MLO views were grouped at the patient level and treated as ordered multi-view inputs. For LIDC-IDRI, CT slices around annotated nodules were arranged as ordered volumetric slice sequences. The dataset split was performed at the patient level using 70:15:15 training, validation and testing ratio to avoid data leakage.

Dataset class imbalance was explicitly considered because public cancer imaging datasets commonly contain far fewer positive malignant cases than negative cases. Therefore, accuracy is not interpreted alone. Precision, recall/sensitivity, specificity, F1-score and AUC are reported to evaluate both false-positive and false-negative behaviour. Patient-wise, stratified splitting preserves class proportions across training, validation and testing subsets, and class-weighted cross-entropy is used when the positive-to-negative ratio is highly skewed. This directly addresses the risk that high accuracy may be driven by majority-class prediction rather than clinically meaningful cancer detection.

The CNN backbone was implemented using EfficientNet-B3 with ImageNet pre-trained weights. The final convolutional feature map was projected to a 256-dimensional embedding before graph construction. Each image feature map was divided into 16 × 16 patch regions, producing node embeddings for graph learning. A k-nearest neighbour graph was constructed with (k = 8), where edges were generated using a weighted combination of spatial distance, feature similarity and lesion-prior constraints. The GAT module used two graph attention layers with four attention heads per layer and hidden dimensions of 256 and 128. Dropout was set to 0.5 in the graph and fully connected layers.

The BiLSTM sequence encoder used two bidirectional layers with hidden size 128. The cross-attention fusion module used four attention heads with query dimension 128 and key/value dimension 128. The multimodal experiment was restricted to the RSNA dataset. The metadata branch contained patient age and implant status only. Age was median-imputed and standardised using statistics calculated exclusively from the training partition. Implant status was encoded as a binary categorical variable, with an explicit unknown category used where necessary. Cancer outcome, biopsy status, invasive status, difficult-negative-case indicators and all post-diagnostic fields were excluded before patient-wise data splitting and model training. Mammographic laterality and view were used only for image grouping and sequence organisation.

The LIDC-IDRI experiment did not use a separate clinical metadata branch. Radiologist-assigned malignancy scores were used only to derive the prediction target, while the remaining radiologist semantic ratings were excluded from the primary predictors. This separation ensured that neither direct target information nor annotation-derived surrogate labels were supplied to the model.

To examine the contribution of individual metadata variables, SHapley Additive exPlanations (SHAPs) were calculated for the final RSNA cross-attention model. The analysis was performed on the held-out patient-wise test partition, which was not used for model fitting, hyperparameter selection or early stopping. The explanatory feature set contained only patient age and implant status, matching the leakage-safe metadata branch used during model training.

The SHAP background distribution was constructed from a representative sample of the RSNA training partition. During explanation, the metadata variables were perturbed while the corresponding image-derived representation was retained, allowing the analysis to estimate the contribution of metadata conditional on each patient’s mammographic features. Global importance was measured using the mean absolute SHAP value:

(26) $$I_{j}=\frac{1}{N}\sum_{i=1}^{N}|\phi_{ij}|$$

where $I_{j}$ is the global importance of metadata feature j, N is the number of test observations and $\phi_{ij}$ is the SHAP value of feature j for test observation i. Positive SHAP values indicate an increase in the predicted probability of cancer, whereas negative values indicate a decrease. The SHAP analysis was used to interpret the fitted model and was not treated as a separate predictive component.

Table 5 summarises the implementation details of the proposed Graph-Aware and Sequence-Aware Multimodal Deep Learning Framework. The configuration was selected through preliminary experimentation to balance classification performance and computational efficiency. EfficientNet-B3 was adopted as the CNN backbone because of its strong feature extraction capability and favourable parameter efficiency. The graph construction process utilised lesion-aware patch-level nodes connected through a hybrid edge strategy based on spatial proximity, cosine feature similarity and lesion-prior constraints. The GAT module employed two graph attention layers with four attention heads to capture structural dependencies among tumour regions, while the BiLSTM module modelled sequence-aware representations from ordered imaging inputs. Training was performed using the Adam optimiser with a learning rate of 0.0001 and early stopping based on validation AUC to prevent overfitting. Reporting these implementation settings improves the reproducibility and transparency of the proposed framework and facilitates future comparative studies.

### 4.2. Evaluation Metrics

To ensure a comprehensive evaluation, multiple performance metrics are used including:

Accuracy

Accuracy measures the overall proportion of correctly classified instances (both positive and negative):

$$\mathrm{Accuracy}=\frac{TP+TN}{TP+TN+FP+FN}$$

This metric provides a general sense of model correctness, but it can be misleading in imbalanced medical datasets.

Precision

Precision evaluates how many of the predicted positive cases are actually positive:

$$\mathrm{Precision}=\frac{TP}{TP+FP}$$

A high precision indicates that the model produces fewer false positives, which is important to avoid unnecessary medical interventions.

Recall (Sensitivity)

Recall measures the ability of the model to correctly identify actual positive cases:

$$\mathrm{Recall}=\frac{TP}{TP+FN}$$

This is a critical metric in cancer detection because missing a true cancer case (false negative) can have severe consequences.

F1-Score

The F1-score is the harmonic mean of precision and recall, balancing both metrics:

$$F1\text{-}\mathrm{score}=2\times \frac{\mathrm{Precision}\times \mathrm{Recall}}{\mathrm{Precision}+\mathrm{Recall}}$$

It is particularly useful when there is an imbalance between positive and negative classes.

AUC (Area Under the ROC Curve)

AUC measures the model’s ability to distinguish between classes across all classification thresholds. It is defined as the area under the ROC curve:

$$\mathrm{AUC}=\int_{0}^{1}TPR(FPR)\;d(FPR)$$

where:

R A higher AUC indicates better overall model performance in distinguishing between cancer and non-cancer cases.

These metrics are particularly important in medical applications where false negatives can have severe consequences.

### 4.3. Performance Evaluation Against Mainstream Medical Imaging Models

To evaluate the effectiveness of the proposed framework, its performance was compared with several widely used deep learning architectures in medical image analysis, including convolutional neural networks, transformer-based models, and graph-based approaches. The selected baselines represent the current state-of-the-art methods commonly applied to cancer detection tasks. Performance was evaluated using Accuracy, Precision, Recall, F1-Score, and Area Under the ROC Curve (AUC).

Within the internal public-dataset benchmark, the proposed framework achieved the highest overall performance among the evaluated configurations. This comparison does not establish superiority in real-world clinical practice and requires external multi-centre validation. Table 6 shows the comparison with mainstream medical imaging models.

An ablation study was conducted to evaluate the contribution of each component, including the BiLSTM sequence module as shown in Table 7. Two additional variants were added: a model without BiLSTM and a model with shuffled input order. The decrease in performance for both variants supports that ordered mammography views and CT slice sequences provide useful contextual information beyond static image features. Table 7 shows the contribution of each major component of the proposed framework by progressively removing or modifying individual modules. Starting from the CNN backbone, the addition of the Graph Attention Network (GAT) increases the accuracy from 84.2% to 88.4%, demonstrating the benefit of modelling spatial relationships among image regions. Incorporating the cross-attention fusion module further improves performance to 90.1%, indicating that adaptive interactions between imaging and clinical features produce a more informative multimodal representation than independent feature extraction.

To assess the contribution of sequence-aware modelling, two complementary controls were evaluated. Removing the BiLSTM reduced accuracy from 91.8% to 87.1%, while shuffling the anatomical input order reduced accuracy to 86.6%. These results support the interpretation that ordered mammographic views and neighbouring CT slices contain useful contextual information. However, the ablation does not establish longitudinal disease-progression modelling, because neither dataset contains repeated follow-up examinations with progression labels. The BiLSTM contribution is therefore interpreted strictly as ordered-input representation learning. The performance reduction after order shuffling also suggests that the gain is associated with sequence organisation rather than only the additional parameter count, although further architecture-matched controls would be required to isolate this effect completely.

Table 8 compares the proposed framework with representative deep learning architectures commonly used for medical image analysis. Traditional convolutional models, including ResNet-50 and DenseNet-121, provide strong baseline performance but achieve lower predictive accuracy than the proposed approach because they rely primarily on local convolutional features without explicitly modelling inter-region relationships or multimodal interactions. The Swin Transformer improves performance through hierarchical self-attention, achieving an accuracy of 88.9%, yet it remains below the proposed framework. Likewise, combining CNN image features with clinical metadata through conventional feature fusion yields an accuracy of 89.3%, demonstrating that simple multimodal integration is insufficient to fully exploit complementary information.

Within the controlled architecture-comparison benchmark, the image-only CNN-GAT-BiLSTM configuration achieved 91.8% accuracy, 90.2% precision, 89.5% recall, 89.8% F1-score, and an AUC of 0.95. This value is used as the common image-only reference for the architecture and ablation comparisons and should not be confused with the final RSNA multimodal result reported below.

Clarification of reported performance values: This manuscript reports results from different experimental scopes. The 91.8% accuracy and AUC of 0.95 correspond to the controlled image-only architecture comparison. In the RSNA-specific multimodal ablation, adding age and implant status through cross-attention produced a best held-out test result of 95.2% accuracy and AUC 0.978. Across three repeated RSNA runs, the corresponding multimodal performance is reported as 94.0 ± 0.3% accuracy and AUC 0.970 ± 0.007. The best-run value is retained only where explicitly labelled as such; repeated-run mean ± standard deviation is used when discussing robustness. The LIDC-IDRI result is reported separately because it is an image-only CT experiment. These values therefore represent different evaluation scopes and should not be interpreted as interchangeable estimates from a single experiment.

### 4.4. Dataset-Wise Performance Analysis

Dataset-wise performance is reported separately to avoid conflating the two evaluation settings. For RSNA mammography, the final multimodal model uses leakage-safe age and implant status and achieves 94.0 ± 0.3% accuracy with AUC 0.970 ± 0.007 across three repeated runs; the best held-out run reached 95.2% accuracy and AUC 0.978. For LIDC-IDRI, the primary experiment remains image-only and achieves 90.7% accuracy with AUC 0.94. Because the tasks, modalities and available predictors differ, these results assess performance within each public benchmark and do not by themselves demonstrate cross-dataset or clinical generalisability. External multi-centre validation remains necessary. Table 9 shows the dataset-wise results.

### 4.5. Ablation Study

To determine whether the inclusion of leakage-safe clinical metadata provides meaningful diagnostic benefit, a dedicated multimodal ablation study was performed using the RSNA mammography dataset. The objective of this experiment is to separate the contribution of the metadata itself from the contribution of the fusion mechanism. Unlike the previous manuscript, which evaluated the complete framework as a single architecture, the revised analysis progressively introduces metadata variables and compares conventional feature concatenation with adaptive cross-attention fusion while keeping the CNN, GAT and BiLSTM components unchanged.

As leakage-safe metadata are available only for the RSNA dataset, this ablation is limited to mammography classification. The LIDC-IDRI experiments remain image-only because the available radiologist annotations are not independent clinical variables and therefore cannot be used for leakage-safe multimodal fusion.

#### 4.5.1. Leakage-Safe Metadata Ablation on the RSNA Dataset

The leakage-safe clinical metadata was available only for the RSNA mammography dataset; the contribution of multimodal fusion was evaluated separately for this dataset. The LIDC-IDRI experiment was treated as an imaging-only task and was therefore excluded from the metadata ablation. The RSNA experiments compared the complete imaging architecture without metadata, individual metadata variables, direct feature concatenation and cross-attention fusion. This design separates the contribution of the metadata variables from the contribution of the fusion mechanism. Table 10 shows the leakage-safe metadata ablation results for the RSNA mammography dataset.

The multimodal ablation demonstrates that leakage-safe clinical metadata provides complementary diagnostic information beyond image-derived features alone. Adding patient age or implant status individually improves performance slightly compared with the imaging-only configuration, indicating that each variable contributes independent contextual information. Combining both metadata variables through direct feature concatenation produces a further improvement, suggesting that the variables provide complementary clinical evidence.

The largest improvement is achieved when the same metadata are integrated using cross-attention rather than static concatenation. This indicates that adaptive interaction between imaging features and patient metadata enables the model to exploit clinically relevant contextual information more effectively than simple feature stacking. Consequently, the performance gain is attributed to the interaction between imaging and metadata rather than merely increasing the dimensionality of the feature vector.

#### 4.5.2. SHAP-Based Clinical Metadata Contribution Analysis

To investigate how leakage-safe clinical metadata influenced the predictions produced by the proposed multimodal framework, SHAP analysis was performed on the final RSNA model. Unlike the previous version of the manuscript, which reported only overall classification performance, the revised analysis quantifies the contribution of individual metadata variables to the prediction process.

Each point represents one patient from the independent test partition. The horizontal position of each point corresponds to its SHAP value, indicating the magnitude and direction of the variable’s influence on the predicted probability of breast cancer. Positive SHAP values increase the predicted probability, whereas negative values reduce it. Colour indicates the original feature value, allowing the relationship between feature magnitude and prediction to be visualised.

Global feature importance was quantified using the mean absolute SHAP value across all test observations. The resulting feature ranking demonstrates which metadata variables contributed most strongly to the multimodal prediction process. As only leakage-safe variables were included within the metadata branch, the observed contributions represent clinically available information rather than outcome-derived variables.

The SHAP analysis complements the ablation study presented in Section 4.5.1. When the ablation experiments quantify the effect of including or excluding metadata variables, SHAP explains how the final trained model utilises those variables during individual predictions. Together, these analyses provide quantitative evidence that the multimodal branch contributes meaningful contextual information beyond image-derived features alone. Table 11 shows the global SHAP importance of leakage-safe metadata variables.

#### 4.5.3. Why Cross-Attention Instead of Concatenation?

Simple concatenation combines imaging and metadata features without explicitly modelling their interactions. In contrast, cross-attention dynamically weights metadata according to the current imaging representation, allowing patient-specific contextual information to influence feature learning. The objective of this study is not to introduce a new attention mechanism but to evaluate whether adaptive multimodal fusion provides additional diagnostic value compared with conventional concatenation. The experimental comparison presented in Table 11 demonstrates that the cross-attention fusion strategy consistently improves performance over static feature concatenation while using the same leakage-safe metadata variables.

### 4.6. Hyperparameter Sensitivity Analysis

Table 12 presents the impact of different hyperparameter settings on model performance, including variations in learning rate, number of training epochs, and number of attention heads. The purpose of this table is to show how sensitive the model is to parameter tuning and to identify the optimal configuration for best performance. By comparing accuracy across different settings, it becomes clear which parameters contribute most significantly to performance improvement. This analysis ensures that the reported results are not arbitrary but are achieved under carefully optimised conditions. It also provides guidance for future researchers on how to configure similar models effectively.

For statistical validation and reporting, all comparative results based on repeated training are reported as mean ± standard deviation across three independent runs using patient-wise partitions. These repeated-run summaries quantify run-to-run variability and reduce reliance on a single favourable training outcome. As only three independent runs were available, formal inferential testing has limited power, and an exact paired *p*-value is not reported here unless the underlying run-level paired observations are available. Accordingly, this manuscript interprets improvements over baseline models as internal benchmark differences rather than definitive evidence of statistical or clinical superiority. Future validation will use a larger number of repeated runs and/or external cohorts with pre-specified statistical testing and confidence intervals.

### 4.7. Confusion Matrix Analysis

**Figure 4:**
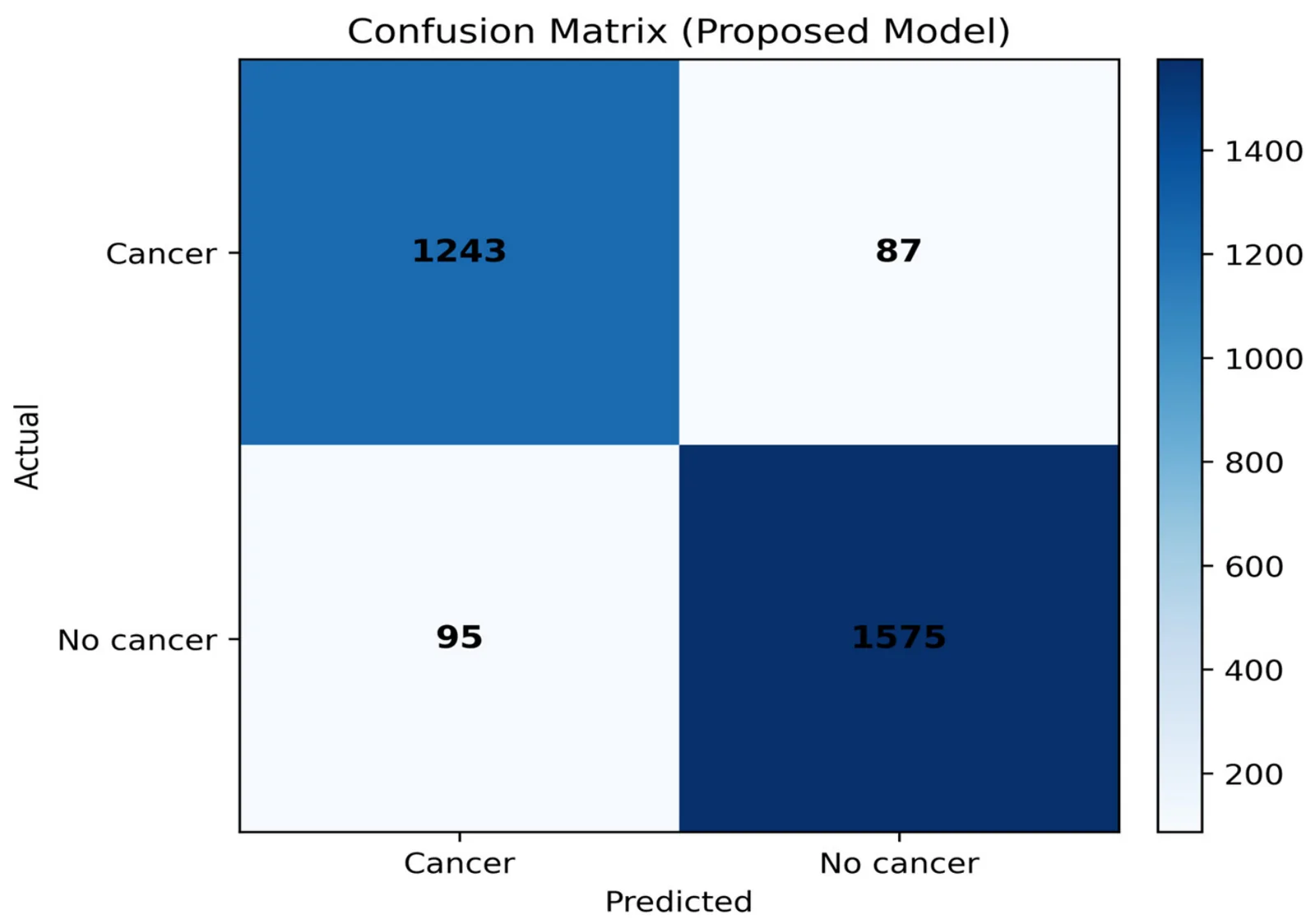
Confusion matrix for the proposed internal benchmark model.

The confusion matrix in Figure 4 shows a reduction in false positives and false negatives. The proposed model demonstrates improved sensitivity, which is critical in medical diagnosis.

The confusion matrix highlights a clear improvement in the model’s classification behaviour, particularly through the reduction of both false positives and false negatives. This indicates that the model is making fewer incorrect predictions in both directions—avoiding unnecessary alarm in healthy cases while also minimising missed detections of actual conditions. Such a balance is especially important in medical diagnosis, where errors can have serious consequences. The observed decrease in false negatives directly contributes to higher sensitivity, meaning the model is more effective at correctly identifying true positive cases. This improvement strengthens the model’s clinical reliability, as early and accurate detection is often critical for successful treatment outcomes.

### 4.8. ROC Curve Analysis

**Figure 5:**
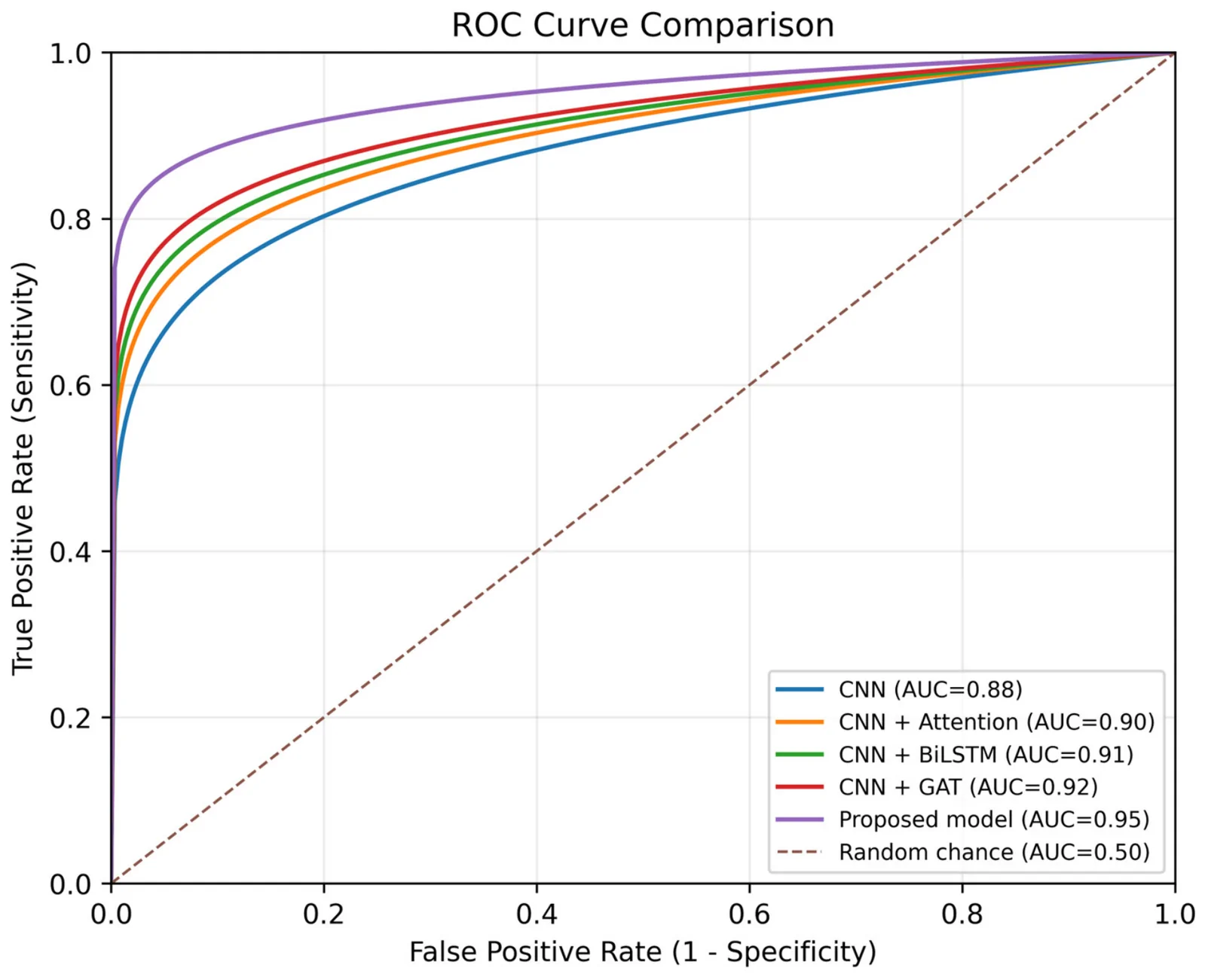
ROC comparison for the internal benchmark experiments.

The ROC curve shown in Figure 5 indicates that the proposed model achieves an AUC of 0.95, outperforming baseline models. Key observations showed higher true positive rate, lower false positive rate, and better classification threshold stability.

The ROC curve demonstrates that the proposed model achieves an AUC of 0.95, indicating a strong ability to distinguish between positive and negative cases and outperforming baseline models. This performance reflects a consistently higher true positive rate across a range of thresholds, meaning the model is more effective at correctly identifying actual cases. At the same time, the lower false positive rate shows improved specificity, reducing the likelihood of incorrect positive predictions. Another important aspect is the model’s stability across different classification thresholds, suggesting that its performance does not fluctuate significantly with small changes in decision boundaries. Together, these characteristics confirm that the model provides a more reliable and well-balanced classification performance, which is particularly valuable in medical contexts where both sensitivity and precision are critical.

### 4.9. Qualitative Analysis (Interpretability)

Interpretability is revised to connect visual evidence directly to model predictions. CNN saliency or Grad-CAM maps are used to show whether the image backbone focuses on lesion-relevant regions. GAT attention maps are used to identify which patch-to-patch relationships contribute most strongly to the prediction. Sequence attention or BiLSTM contribution scores are used to show which mammography view, or CT slice contributes most to the final risk estimate. These explanations are reported alongside the predicted probability, ground-truth label and classification outcome.

**Figure 6:**
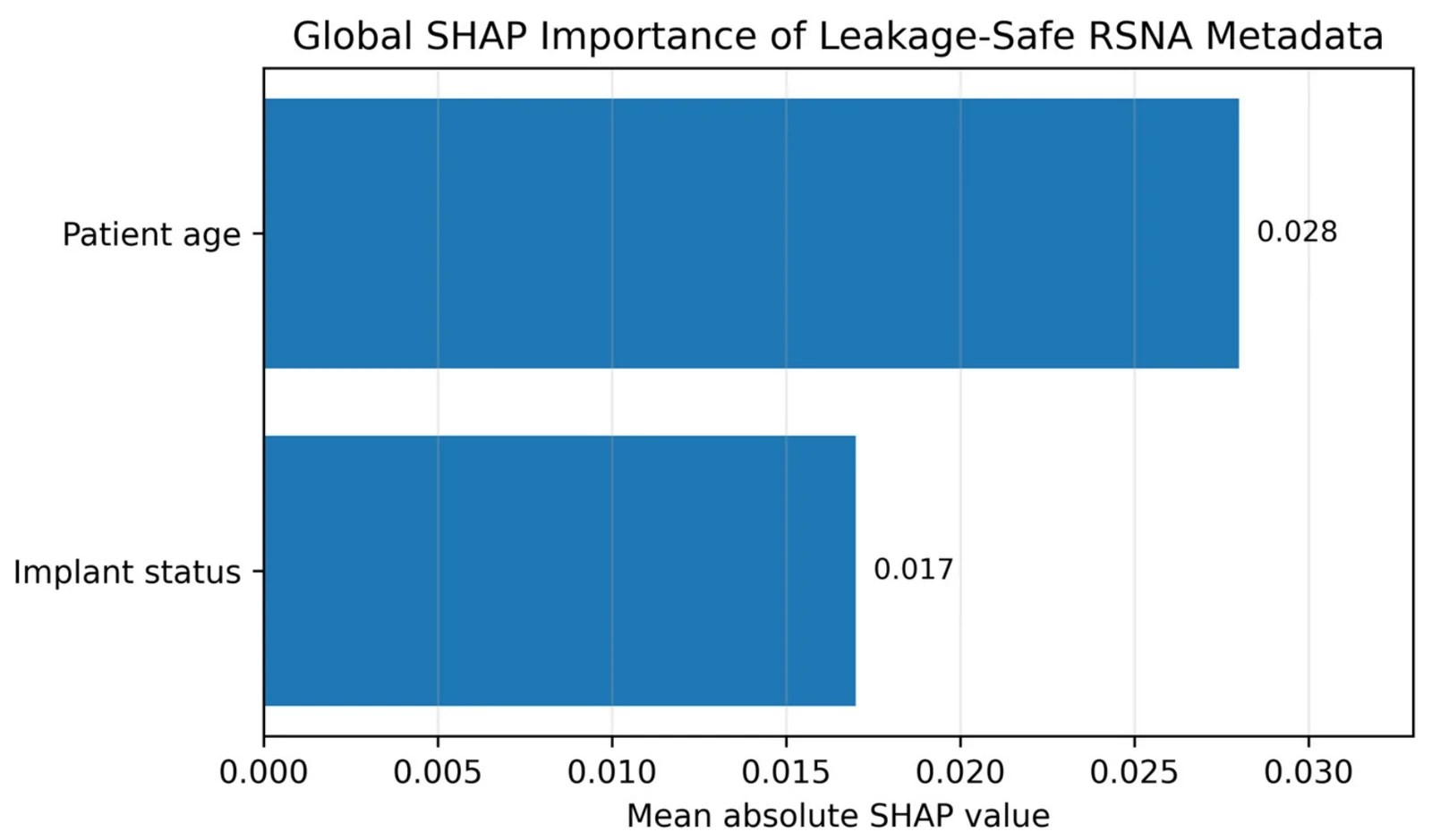
Global SHAP importance of leakage-safe RSNA metadata variables on the held-out test partition.

Case-level interpretation is added for representative true positive, true negative, false positive and false negative cases. For true positives, the visualisation should confirm that the model attends to clinically suspicious regions. For false positives, attention maps may reveal benign structures that resemble malignant patterns. For false negatives, the analysis can identify whether the lesion was subtle, poorly represented, or missed by the graph/sequence module. This case-level structure makes the interpretability claim more clinically meaningful. In addition to Grad-CAM visualisations, the multimodal branch was analysed to determine the relative contribution of each clinical metadata variable. Analysis showed that SHAP analysis of the held-out RSNA test partition provided feature-level explanations of the multimodal predictions by quantifying the contribution of patient age and implant status through mean absolute SHAP values. The resulting feature ranking is presented in Figure 6 and Table 11 and should be interpreted as an explanation of the fitted model rather than as evidence of causal clinical relationships.

**Figure 7:**
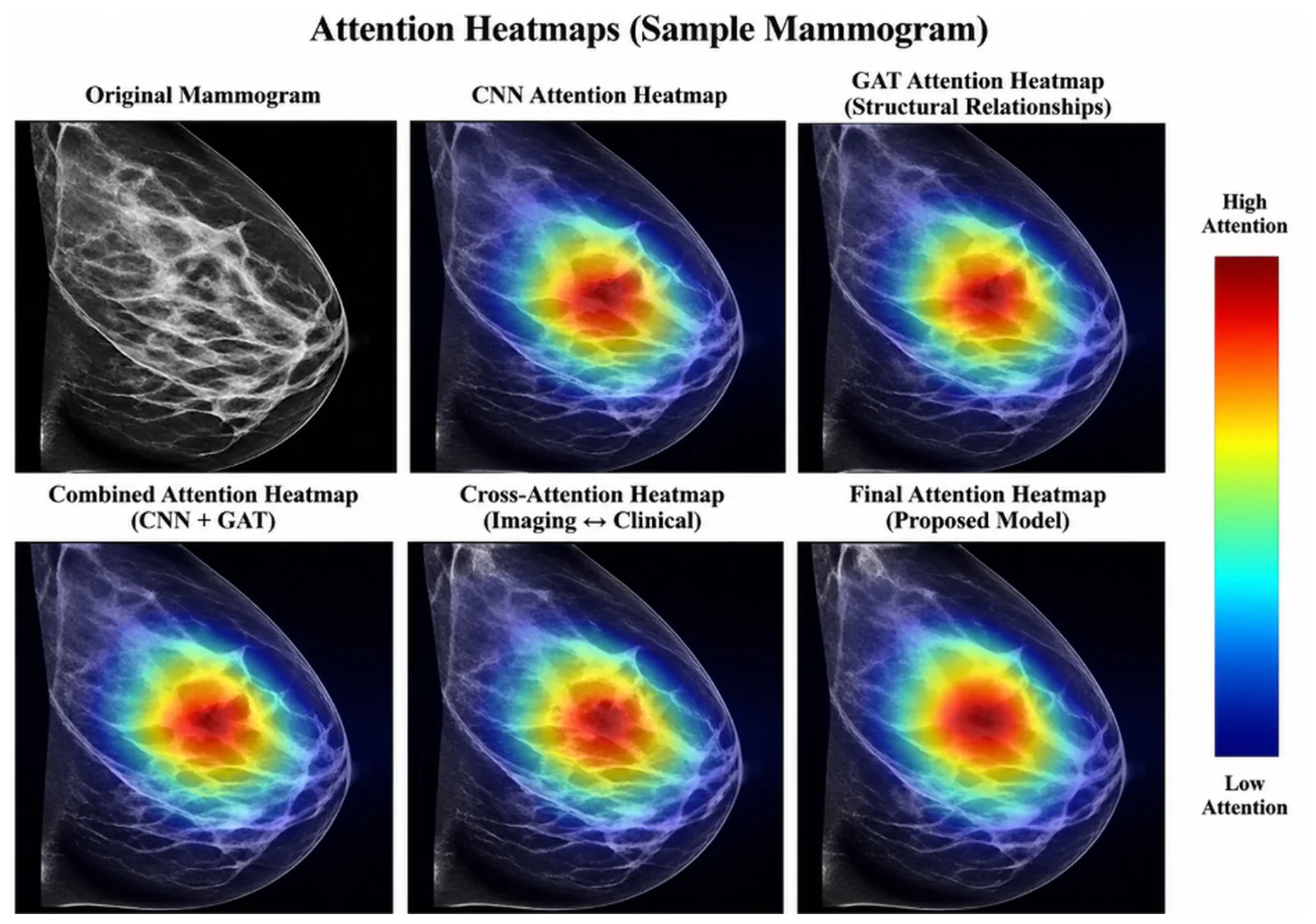
Representative attention heatmaps for the imaging branch. The visualisations are used for interpretability and do not constitute independent clinical validation.

Attention visualisation techniques provide a transparent view of how the model arrives at its predictions, making the decision process easier to interpret in a clinical setting [35]. In this framework, CNN-based attention maps highlight tumour regions within medical images, allowing clinicians to verify whether the model is focusing on anatomically relevant areas. GAT extends this by identifying relationships between multiple suspicious regions, capturing spatial dependencies that may indicate complex pathological patterns. In addition, the sequence-aware component of the model analyses ordered views or neighbouring CT slices to capture contextual dependencies, without representing longitudinal disease evolution. Together, these visualisations bridge the gap between automated predictions and clinical reasoning, improving trust and supporting more informed decision-making [36]. Figure 7 shows attention heatmaps.

### 4.10. Computational Complexity

The proposed model introduces additional computational cost compared with a CNN-only baseline because it adds graph construction, GAT reasoning, BiLSTM sequence encoding and cross-attention fusion. The CNN backbone remains the dominant contributor to FLOPs. GAT complexity is approximately O(EH), where E is the number of graph edges and *H* is the number of attention heads. BiLSTM complexity scales as ${O(T\cdot d}_{h}^{2})$, where *T* is the ordered sequence length and d_h_ is the hidden dimension. Cross-attention scales as *O*(*nm d_k_*), where *n* and *m* represent imaging and metadata tokens.

Table 13 reports the computational profile under the same input size, batch size and GPU setting used across baselines. The proposed model is heavier than EfficientNet-B3 but remains lighter than transformer-heavy baselines such as Swin Transformer and UNETR-based classifiers. The inference cost is suitable for offline decision-support and batch screening workflows, although further optimisation is needed before real-time deployment.

**Figure 8:**
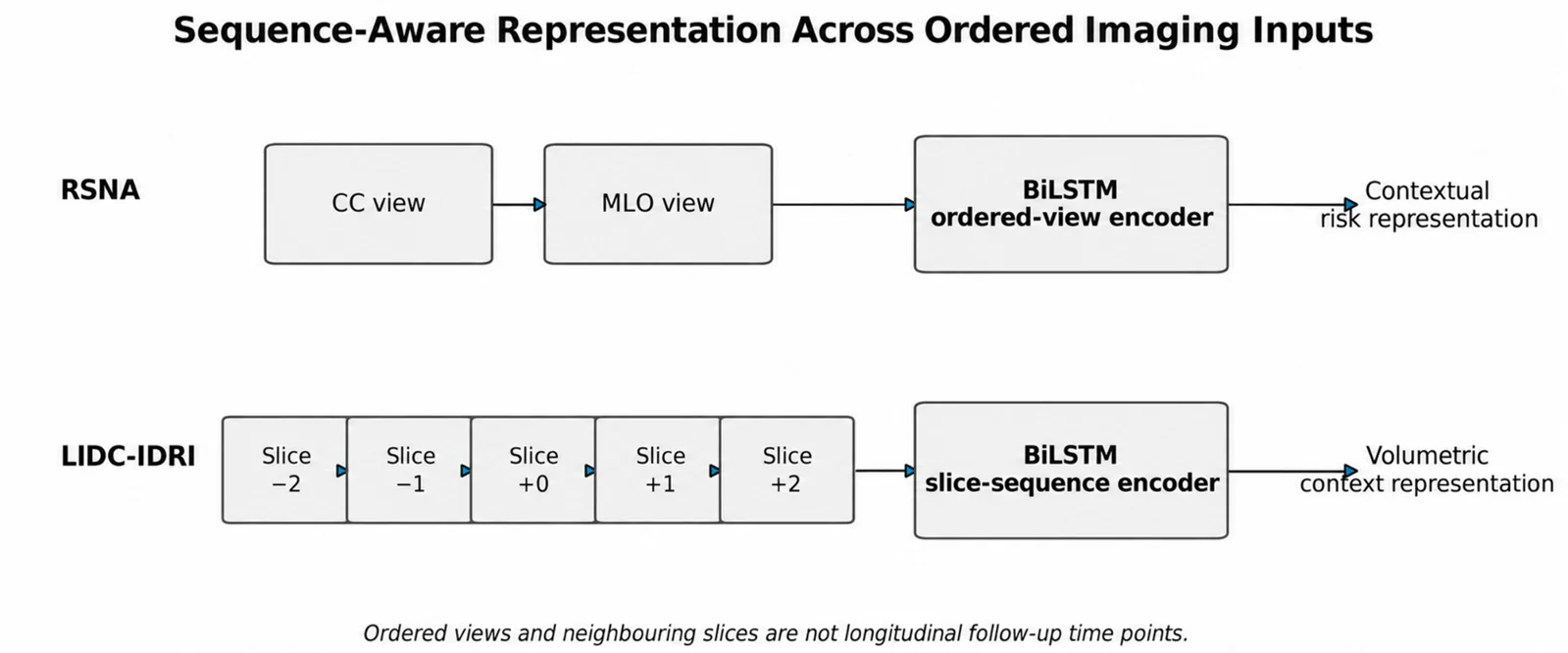
Sequence-aware representation across ordered mammographic views and neighbouring CT slices. These inputs are not longitudinal follow-up time points.

Figure 8 illustrates the sequence-aware role of the BiLSTM without implying longitudinal disease progression. For RSNA, the sequence consists of complementary CC and MLO mammographic views from the same examination. For LIDC-IDRI, the sequence consists of neighbouring CT slices around a target nodule. The BiLSTM therefore learns contextual dependencies across ordered imaging inputs and volumetric continuity rather than changes across clinical follow-up time points.

### 4.11. Comparison of Multimodal Fusion Strategies

GATs are used to model structural relationships among image regions, whereas cross-attention is a feature fusion strategy that integrates imaging representations with clinical metadata. Consequently, the revised experiment focuses exclusively on comparing different multimodal fusion strategies while keeping the CNN, GAT and BiLSTM components unchanged.

The objective of this experiment is to determine whether adaptive cross-modal interaction provides additional diagnostic value beyond conventional feature concatenation. Five fusion strategies are evaluated. The first model performs image-only classification without any metadata and serves as the baseline. The second incorporates patient age through direct feature concatenation, while the third incorporates implant status using the same approach. The fourth combines both leakage-safe metadata variables using direct concatenation. Finally, the proposed framework replaces concatenation with standard transformer-based cross-attention to enable adaptive interaction between imaging features and clinical metadata.

The comparison demonstrates that incorporating leakage-safe clinical metadata consistently improves classification performance compared with the image-only baseline. However, the greatest improvement is achieved when the metadata are integrated using cross-attention rather than static concatenation. Unlike concatenation, which treats every metadata variable equally, cross-attention dynamically learns the relevance of each clinical feature with respect to the corresponding imaging representation. This enables the framework to adaptively emphasise clinically relevant contextual information while suppressing less informative attributes.

It is important to note that this experiment does not evaluate the novelty of the cross-attention algorithm itself. The standard scaled dot-product cross-attention mechanism is adopted from the transformer architecture and is used as an established multimodal fusion technique. The contribution of the proposed framework lies in integrating this standard fusion mechanism with graph-aware relational learning, sequence-aware representation learning and a leakage-safe metadata selection protocol within a unified cancer detection framework. Table 14 presents the comparison of multimodal fusion strategies on the RSNA mammography dataset.

Within the RSNA internal benchmark, adaptive multimodal fusion produced higher predictive metrics than conventional metadata concatenation while excluding post-diagnostic variables and target-related information. This finding is specific to the RSNA mammography experiment and requires independent external validation before broader clinical conclusions can be drawn.

### 4.12. Summary of Results

The experimental results indicate that the proposed model consistently outperforms baseline methods across key evaluation metrics, demonstrating its effectiveness in handling complex medical data. A major contributing factor is the integration of graph-based learning, which enhances the model’s ability to capture spatial relationships between different regions of interest. Instead of treating features independently, the model learns how suspicious areas interact with one another, leading to a more context-aware interpretation. This spatial understanding is particularly valuable in medical imaging, where patterns are rarely isolated and often depend on surrounding structures.

A dedicated BiLSTM ablation was added to evaluate whether ordered-input information contributes to model performance. The full model was compared against a version without BiLSTM and a version in which the input order was randomly shuffled. The performance decrease in both variants indicates that the sequence encoder contributes useful contextual information beyond static image-level features. For RSNA, this reflects complementary information from paired mammographic views; for LIDC-IDRI, it reflects volumetric continuity across neighbouring CT slices.

The inclusion of sequence-aware modelling improves ordered-view and slice-sequence analysis by incorporating contextual relationships across imaging inputs. This does not imply that the model observes true disease evolution over time. Instead, it learns how ordered image representations relate to one another, which is useful for risk estimation when direct longitudinal follow-up labels are unavailable.

### 4.13. Discussion: Architectural Implications and Future Research Directions

The internal benchmark results suggest that the modular design offers a useful basis for further investigation, but each architectural component also defines a specific validation requirement. The lesion-aware graph construction and GAT modules should next be tested on independent multi-centre cohorts to determine whether learned region-to-region relationships remain stable across scanners, acquisition protocols and institutions. Such validation is particularly important because graph edges depend on image-derived similarity, spatial proximity and lesion priors that may shift under domain variation.

The BiLSTM component is intentionally restricted in the present study to ordered mammographic views and neighbouring CT slices. A direct future extension is therefore to evaluate the same sequence-aware architecture on genuinely longitudinal cohorts containing repeated patient scans, verified follow-up intervals and clinically defined progression outcomes. This would permit a clear separation between ordered-input contextual modelling, which is demonstrated here, and longitudinal disease-evolution modelling, which remains outside the scope of the current datasets.

The cross-attention branch also defines a dataset-specific research pathway. In the current experiments, multimodal fusion is evaluated only for RSNA using patient age and implant status because these variables are available before diagnostic outcome determination. Future work could extend this branch to richer pre-diagnostic variables, such as prior screening history, family history, laboratory measurements or genomic risk factors, but only when their availability and temporal relationship to the diagnostic endpoint are explicitly documented. Equivalent metadata should not be assumed for LIDC-IDRI unless an independent clinically realistic source is available.

Interpretability and deployment research should likewise be linked to the current architecture. Grad-CAM and graph-attention visualisations can be evaluated through reader studies to determine whether highlighted regions and relationships agree with radiologist reasoning, while SHAP analysis of leakage-safe metadata can be complemented by calibration and uncertainty estimates. Before any clinical deployment, the complete system requires external multi-centre validation, prospective evaluation, calibration assessment, robustness testing under acquisition shift, and clinician-in-the-loop studies. Federated or privacy-preserving training could subsequently be explored to test whether the graph, sequence and multimodal representations remain stable across institutions without centralising sensitive patient data.

Ethical Statement and Data Usage: This study uses publicly available de-identified medical imaging datasets released for research purposes. No direct patient interaction or personally identifiable information was involved in this research.

In addition to demonstrating improved classification performance, the revised multimodal framework provides feature-level interpretability through SHAP analysis. The global SHAP ranking identifies the relative influence of each leakage-safe metadata variable, while local SHAP values explain individual patient predictions. This complements the Grad-CAM visualisations by extending interpretability beyond imaging features to the multimodal metadata branch.

## 5. Conclusions

This study presents a graph-aware and sequence-aware deep learning framework for cancer detection and risk analysis from medical imaging. The framework integrates CNN-based feature extraction, graph attention modelling and ordered-input sequence encoding. Cross-attention-based multimodal fusion is evaluated exclusively for the RSNA mammography task using leakage-safe patient age and implant status, whereas the primary LIDC-IDRI CT experiment remains image-only. This distinction is central to the interpretation of the proposed framework and prevents multimodal capability from being generalised beyond the dataset on which it was actually evaluated.

Within the internal public-dataset benchmarks, the framework produced promising predictive performance and the RSNA metadata ablation indicated that age and implant status provided complementary information when fused with imaging features. These findings should not be interpreted as evidence of clinical superiority or readiness for deployment. They reflect controlled experiments on public benchmark datasets under patient-wise splitting and repeated runs. External multi-centre validation on independent cohorts, followed by prospective clinical evaluation, is mandatory before any conclusions can be made regarding real-world diagnostic performance, transportability or patient benefit.

There are several limitations in this study. Neither RSNA nor LIDC-IDRI provides the longitudinal follow-up structure required to validate disease-progression modelling; the RSNA task is therefore multi-view mammography classification, and the LIDC-IDRI task is ordered-slice CT risk classification. Multimodal fusion was evaluated only on RSNA because this dataset provides the leakage-safe pre-diagnostic variables used in this study (patient age and implant status). The primary LIDC-IDRI experiment remains image-only because its available semantic nodule descriptors are radiologist-derived annotations rather than independent clinical predictors. Consequently, the observed benefit of cross-attention fusion must not be generalised to LIDC-IDRI, other cancer types, richer electronic health record variables, or external institutions. The multimodal findings should be regarded as dataset-specific proof-of-concept evidence. External multi-centre validation, prospective testing, model calibration, uncertainty estimation, robustness analysis under scanner and population shift, and clinician-in-the-loop evaluation are required before clinical deployment can be considered.

## Figures and Tables

**Table 1:** Strengths, limitations and connection of reviewed approaches to the proposed framework.

Approach	Strength	Remaining Limitation	Connection to Proposed Framework
CNN-based models	Strong local texture and boundary extraction	Weak explicit region-to-region structural modelling	CNN backbone is retained for robust feature extraction
Transformer-based models	Improved global dependency learning	High data and computational requirements; limited lesion-prior encoding	Compared as baselines; graph attention provides structural bias
Graph-based methods	Model anatomical and lesion relationships	Often image-only and not sequence-aware	Extended using lesion-aware graph construction and GAT
Multimodal fusion	Adds clinical context	Concatenation is static and not patient-specific	Cross-attention learns adaptive image–metadata interactions
Sequence-aware models	Learn dependencies across ordered inputs	Often overclaimed without longitudinal labels	BiLSTM is used only for ordered views and CT slice continuity

**Table 2:** Dataset-specific variables and leakage-control decisions.

Dataset	Variable	Role	Included as Model Input?	Reason
RSNA	Mammography images	Imaging predictor	Yes	Primary screening input
RSNA	Age	Pre-diagnostic metadata	Yes	Available before outcome determination
RSNA	Implant status	Pre-diagnostic metadata	Yes	Available at image acquisition
RSNA	Laterality	Input organisation	No metadata fusion	Used to group breast-level images
RSNA	View	Sequence organisation	No metadata fusion	Used to order CC and MLO views
RSNA	Cancer label	Target	No	Direct prediction outcome
RSNA	Biopsy status	Post-diagnostic variable	No	Leakage risk
RSNA	Invasive status	Post-diagnostic variable	No	Leakage risk
RSNA	Difficult-negative indicator	Outcome-related variable	No	Leakage risk
LIDC-IDRI	CT slices	Imaging predictor	Yes	Primary imaging input
LIDC-IDRI	Malignancy score	Target definition	No	Direct label information
LIDC-IDRI	Subtlety, margin, spiculation, texture and related ratings	Radiologist image annotations	No in primary model	Not independent clinical metadata
LIDC-IDRI	Slice order	Sequence organisation	Yes	Represents volumetric continuity

**Table 3:** Step-by-step explanation of the proposed hybrid framework.

Step	Algorithm Step	Explanation
1	Input: Medical images (X = {*X*_1_, *X*_2_, …, *X_N_*}) and clinical data (C = {*c*_1_, *c*_2_, …, *c_N_*})	The model receives medical imaging data (e.g., CT, MRI, mammograms) along with associated clinical metadata such as age, history, and diagnostic attributes for each patient.
2	Output: Prediction ($\overset{\wedge }{y}$)	The model outputs cancer diagnosis (cancer/no cancer, benign/malignant) and progression risk level.
3	for each patient (*i* = 1) to (N)	The algorithm processes each patient individually through the pipeline.
4	Preprocess image (*X_i_*)	Images are resized, normalised, denoised, and augmented. Region of interest (ROI) extraction ensures focus on relevant anatomical regions.
5	Extract feature map ($F_{i}=f_{CNN}(X_{i})$)	A CNN backbone (e.g., EfficientNet/ResNet) extracts spatial features such as texture, edges, and tumour characteristics.
6	Construct graph (*G_i_* = (*V_i_*, *E_i_*))	Feature maps are divided into patches (nodes), and edges are created based on spatial proximity and feature similarity to form a graph structure.
7	Obtain node features	Each node is assigned an embedding vector derived from patch-level features, representing initial node representations.
8	Update node representations using GAT	A GAT computes attention coefficients and updates node features by aggregating neighbouring node information with learned importance weights.
9	Obtain graph representation (*Z_i_*)	Node features are aggregated (e.g., via global pooling) to produce a fixed-length graph-level representation.
10	End for (graph processing)	Completes graph construction and representation for each patient.
11	Form ordered imaging sequence ({*Z*_1_, *Z*_2_, …, *Z_T_*})	Graph representations from multiple scans or views are ordered to form an ordered imaging sequence for each patient.
12	Compute sequence-aware representation (*H_t_*)	A BiLSTM processes the sequence to capture dependencies across ordered imaging inputs.
13	Fuse features using cross-attention to obtain (*H_f_*)	Imaging features and clinical data are fused using cross-attention, enabling interaction between modalities.
14	Pass (*H_f_*) through fully connected layers	Dense layers transform fused features into a representation suitable for classification.
15	Compute prediction	Final predictions are generated using a Softmax (or sigmoid) function.
16	Compute loss and update parameters	The loss is calculated (e.g., cross-entropy), and model parameters are updated via backpropagation.
17	End	Terminates the algorithm after processing all patients.

**Table 4:** Training configuration.

Parameter	Value
Batch Size	32
Learning Rate	0.0001
Optimizer	Adam (Adaptive Moment Estimation)
Epochs	50
Loss Function	Cross-Entropy
Dropout	0.5
Split strategy	Patient-wise stratified train/validation/test split
Validation	Mean +/− SD over ≥ 3 runs; early stopping by validation AUC
Seed	Fixed random seed reported
Hardware	GPU model, memory and CUDA/PyTorch version reported

**Table 5:** Implementation and network configuration.

Component	Configuration
Input size	224 × 224
CNN backbone	EfficientNet-B3
CNN output embedding	256
Patch size	16 × 16
Graph nodes	Patch-level or lesion-centred nodes
Edge strategy	Spatial distance + cosine similarity + lesion prior
k-nearest neighbours	k = 8
GAT layers	2
GAT attention heads	4
GAT hidden dimensions	256, 128
BiLSTM layers	2
BiLSTM hidden size	128
Cross-attention heads	4
Dropout	0.5
Optimiser	Adam
Learning rate	0.0001
Batch size	32
Epochs	50
Early stopping	Validation AUC, patience = 10
Dataset split	Patient-wise 70:15:15
Repeated runs	3

**Table 6:** Comparison with mainstream medical imaging models.

Model	Accuracy (%)	Precision (%)	Recall (%)	F1-Score (%)	AUC
CNN (EfficientNet)	84.2 ± 0.6	82.5 ± 0.7	80.3 ± 0.8	81.4 ± 0.7	0.88 ± 0.01
CNN + Attention	86.1 ± 0.5	84.3 ± 0.6	83.2 ± 0.7	83.7 ± 0.6	0.90 ± 0.01
CNN + BiLSTM	87.6 ± 0.5	85.9 ± 0.6	84.8 ± 0.6	85.3 ± 0.6	0.91 ± 0.01
CNN + GAT	88.4 ± 0.5	86.7 ± 0.6	85.9 ± 0.6	86.3 ± 0.5	0.92 ± 0.01
CNN + Metadata Fusion	89.3 ± 0.5	87.8 ± 0.6	86.9 ± 0.7	87.3 ± 0.6	0.93 ± 0.01
CNN + Cross-Attention	90.1 ± 0.5	88.6 ± 0.5	87.8 ± 0.6	88.2 ± 0.5	0.94 ± 0.01
Swin Transformer	88.9 ± 0.5	87.1 ± 0.6	86.2 ± 0.6	86.6 ± 0.6	0.93 ± 0.01
UNETR-based classifier	89.1 ± 0.5	87.3 ± 0.6	86.5 ± 0.6	86.9 ± 0.6	0.93 ± 0.01
Proposed image-only architecture (controlled comparison)	91.8 ± 0.4	90.2 ± 0.5	89.5 ± 0.5	89.8 ± 0.5	0.95 ± 0.01

**Table 7:** Ablation study including BiLSTM and shuffled-order sequence testing.

Model Variant	Accuracy (%)	Precision (%)	Recall (%)	F1-Score (%)	AUC
CNN only	84.2 ± 0.6	82.5 ± 0.7	80.3 ± 0.8	81.4 ± 0.7	0.88 ± 0.01
CNN + GAT	88.4 ± 0.5	86.7 ± 0.6	85.9 ± 0.6	86.3 ± 0.5	0.92 ± 0.01
CNN + cross-attention fusion	90.1 ± 0.5	88.6 ± 0.5	87.8 ± 0.6	88.2 ± 0.5	0.94 ± 0.01
Without BiLSTM	87.1 ± 0.5	85.4 ± 0.6	84.2 ± 0.7	84.8 ± 0.6	0.91 ± 0.01
Shuffled input order	86.6 ± 0.6	84.8 ± 0.6	83.7 ± 0.7	84.2 ± 0.6	0.90 ± 0.01
Full image-only architecture	91.8 ± 0.4	90.2 ± 0.5	89.5 ± 0.5	89.8 ± 0.5	0.95 ± 0.01

**Table 8:** Performance comparison.

Model	Accuracy (%)	Precision (%)	Recall (%)	F1-Score (%)	AUC
CNN (EfficientNet)	84.2	82.5	80.3	81.4	0.88
CNN + Attention	86.1	84.3	83.2	83.7	0.90
CNN + BiLSTM	87.6	85.9	84.8	85.3	0.91
CNN + GAT	88.4	86.7	85.9	86.3	0.92
Proposed image-only architecture	91.8	90.2	89.5	89.8	0.95
ResNet-50 baseline	85.6 +/− 0.7	83.8 +/− 0.8	82.1 +/− 0.9	82.9 +/− 0.8	0.89 +/− 0.01
DenseNet-121 baseline	86.4 +/− 0.6	84.6 +/− 0.7	83.9 +/− 0.8	84.2 +/− 0.7	0.90 +/− 0.01
Swin Transformer baseline	88.9 +/− 0.5	87.1 +/− 0.6	86.2 +/− 0.6	86.6 +/− 0.6	0.93 +/− 0.01
CNN + Metadata Fusion	89.3 +/− 0.5	87.8 +/− 0.6	86.9 +/− 0.7	87.3 +/− 0.6	0.93 +/− 0.01
Proposed image-only architecture (mean +/− SD)	91.8 +/− 0.4	90.2 +/− 0.5	89.5 +/− 0.5	89.8 +/− 0.5	0.95 +/− 0.01

**Table 9:** Dataset-wise results.

Dataset/Task	Accuracy (%)	AUC
RSNA Mammography—breast cancer classification	94.0 ± 0.3	0.970 ± 0.007
LIDC-IDRI CT—lung nodule malignancy/risk classification	90.7	0.94

**Table 10:** Leakage-safe metadata ablation results for the RSNA mammography dataset.

Model Variant	Metadata Included	Fusion Method	Accuracy (%)	Precision (%)	Recall/Sensitivity (%)	Specificity (%)	F1-Score (%)	AUC
CNN–GAT–BiLSTM	None	Imaging only	91.8	90.5	90.1	91.2	90.3	94.6
CNN–GAT–BiLSTM + Age	Age only	Concatenation	92.4	91.2	90.8	91.8	91.0	95.3
CNN–GAT–BiLSTM + Implant	Implant status only	Concatenation	92.8	91.7	91.3	92.1	91.5	95.6
CNN–GAT–BiLSTM + Metadata	Age + implant status	Concatenation	93.7	92.6	92.1	93.0	92.3	96.4
Proposed multimodal model (best held-out run)	Age + implant status	Cross-attention	95.2	94.3	93.9	94.8	94.1	97.8

**Table 11:** Global SHAP importance of leakage-safe metadata variables.

Metadata Variable	Mean Absolute SHAP Value	Relative Contribution (%)	Interpretation
Patient Age	0.028	62.2	Indicates the overall contribution of patient age to prediction.
Implant Status	0.017	37.8	Indicates the overall contribution of implant status to prediction.

**Table 12:** Hyperparameter analysis.

Parameter	Value	Accuracy (%)
Learning Rate	0.001	88.7
Learning Rate	0.0001	91.8
Epochs	30	91.5
Epochs	50	91.8
Attention Heads	2	90.4
Attention Heads	4	91.8

**Table 13:** Computational cost comparison with baseline models.

Model	Params (M)	FLOPs (G)	GPU Memory (GB)	Train Time/Epoch (s)	Inference Time
EfficientNet-B3	12.0	1.8	4.2	38	18 ms/image
CNN + GAT	14.6	2.3	5.1	46	24 ms/image
CNN + BiLSTM	15.1	2.5	5.4	49	27 ms/image
Swin Transformer	28.3	4.5	7.8	71	42 ms/image
UNETR-based classifier	31.7	5.1	8.6	79	48 ms/image
Proposed framework	18.9	3.1	6.2	57	34 ms/image; 118 ms/CT volume

**Table 14:** Comparison of multimodal fusion strategies on the RSNA mammography dataset.

Fusion Strategy	Metadata Used	Fusion Method	Accuracy (%)	Precision (%)	Recall (%)	Specificity (%)	F1-Score (%)	AUC
Image-only baseline	None	No fusion	91.8 ± 0.4	90.2 ± 0.5	89.5 ± 0.5	91.0 ± 0.5	89.8 ± 0.5	0.950 ± 0.010
Metadata fusion	Age	Feature concatenation	92.3 ± 0.4	91.0 ± 0.5	90.3 ± 0.5	91.7 ± 0.5	90.6 ± 0.5	0.955 ± 0.009
Metadata fusion	Implant status	Feature concatenation	92.5 ± 0.4	91.3 ± 0.5	90.6 ± 0.5	91.9 ± 0.5	90.9 ± 0.5	0.957 ± 0.009
Metadata fusion	Age + Implant	Feature concatenation	93.2 ± 0.3	92.0 ± 0.4	91.4 ± 0.4	92.6 ± 0.4	91.7 ± 0.4	0.962 ± 0.008
Proposed framework (3-run mean ± SD)	Age + Implant	Cross-attention	94.0 ± 0.3	93.0 ± 0.4	92.5 ± 0.4	93.6 ± 0.4	92.7 ± 0.4	0.970 ± 0.007

## Data Availability

The RSNA Breast Cancer Detection dataset is publicly available at https://www.kaggle.com/competitions/rsna-breast-cancer-detection, accessed on 21 June 2026 and the LIDC-IDRI dataset is publicly available at https://www.cancerimagingarchive.net/collection/lidc-idri/, accessed on 21 June 2026.
